# Systemic exposure to aflibercept after intravitreal injection in premature neonates with retinopathy of prematurity: results from the FIREFLEYE randomized phase 3 study

**DOI:** 10.1038/s41433-023-02919-9

**Published:** 2024-01-10

**Authors:** Andreas Stahl, Noriyuki Azuma, Wei-Chi Wu, Domenico Lepore, Emine Sukgen, Hidehiko Nakanishi, Jan Mazela, Sergio Leal, Alexander Pieper, Sarah Schlief, Thomas Eissing, Kenneth C. Turner, An Zhao, Julia Winkler, Joachim Höchel, Evra Köfüncü, Torsten Zimmermann, on behalf of the FIREFLEYE Study Group

**Affiliations:** 1 Department of Ophthalmology, University Medicine Greifswald, Greifswald, Germany; 2 Department of Ophthalmology and Laboratory for Visual Science, National Centre for Child Health and Development, Tokyo, Japan; 3 Department of Developmental and Regenerative Biology, Medical Research Institute, Tokyo Medical and Dental University, Tokyo, Japan; 4 Department of Ophthalmology, Linkou Chang Gung Memorial Hospital, and College of Medicine, Chang Gung University, Taoyuan, Taiwan; 5 Department of Geriatrics and Neuroscience, Catholic University of the Sacred Heart, A. Gemelli Foundation IRCCS, Rome, Italy; 6 Department of Ophthalmology, Health Science University, Adana City Training and Research Hospital, Adana, Turkey; 7 Research and Development Center for New Medical Frontiers, Department of Advanced Medicine, Division of Neonatal Intensive Care Medicine, Kitasato University School of Medicine, Sagamihara, Japan; 8 Department of Neonatology, Poznan University of Medical Sciences, Poznan, Poland; 9 Bayer Consumer Care AG, Basel, Switzerland; 10 Chrestos Concept GmbH & Co., Essen, Germany; 11 Bayer AG, Berlin, Germany; 12 Bayer AG, Leverkusen, Germany; 13 Regeneron Pharmaceuticals Inc., Tarrytown, NY USA; 14 OCCAMS, Amstelveen, the Netherlands

**Keywords:** Retinal diseases, Outcomes research, Paediatrics, Adverse effects

## Abstract

**Background:**

There are no data on pharmacokinetics, pharmacodynamics, and immunogenicity of intravitreal aflibercept in preterm infants with retinopathy of prematurity (ROP). FIREFLEYE compared aflibercept 0.4 mg/eye and laser photocoagulation in infants with acute-phase ROP requiring treatment.

**Methods:**

Infants (gestational age ≤32 weeks or birthweight ≤1500 g) with treatment-requiring ROP in ≥1 eye were randomized 2:1 to receive aflibercept 0.4 mg or laser photocoagulation at baseline in this 24-week, randomized, open-label, noninferiority, phase 3 study. Endpoints include concentrations of free and adjusted bound aflibercept in plasma, pharmacokinetic/pharmacodynamic exploration of systemic anti-vascular endothelial growth factor effects, and immunogenicity.

**Results:**

Of 113 treated infants, 75 received aflibercept 0.4 mg per eye at baseline (mean chronological age: 10.4 weeks), mostly bilaterally (71 infants), and with 1 injection/eye (120/146 eyes). Concentrations of free aflibercept were highly variable, with maximum concentration at day 1, declining thereafter. Plasma concentrations of adjusted bound (pharmacologically inactive) aflibercept increased from day 1 to week 4, decreasing up to week 24. Six infants experienced treatment-emergent serious adverse events within 30 days of treatment; aflibercept concentrations were within the range observed in other infants. There was no pattern between free and adjusted bound aflibercept concentrations and blood pressure changes up to week 4. A low-titer (1:30), non-neutralizing, treatment-emergent anti-drug antibody response was reported in 1 infant, though was not clinically relevant.

**Conclusions:**

24-week data suggest intravitreal aflibercept for treatment of acute-phase ROP is not associated with clinically relevant effects on blood pressure, further systemic adverse events, or immunogenicity.

**ClinicalTrials.gov Identifier:**

NCT04004208.

## Introduction

Retinopathy of prematurity (ROP) is a vasoproliferative retinal disorder in preterm infants. Key risk factors are low gestational age, low birthweight, and postnatal oxygen supplementation [1, 2]. Typically, ROP is mild and patients recover spontaneously. However, some patients develop severe ROP, which can result in vision impairment or loss due to retinal detachment [3]. Thus, treatment must be timely once the need is identified. Vascular endothelial growth factor (VEGF) is an important angiogenic factor during embryonic vascular development [4]. Hypoxia-induced upregulation of VEGF and the role of VEGF in exacerbating vascular proliferation in the vasoproliferative phase of ROP are well known and have led to the increasing use of anti-VEGF agents in ROP [5–7]. Intravitreal aflibercept has been investigated in the FIREFLEYE study in infants with ROP, where it was administered as a 0.4 mg dose per eye and compared with laser photocoagulation [8]. Noninferiority of intravitreal aflibercept was not met statistically; however, the study showed a clinically meaningful response to aflibercept well within the expected range compared with other randomized clinical trials of anti-VEGF agents in ROP [8].

Aflibercept is a recombinantly produced fusion protein consisting of ligand-binding portions of the human VEGF receptor extracellular domains fused to the crystallizable fragment (Fc) region of human immunoglobulin G1 (IgG1), binds monomerically (1:1) with VEGF, and is approved for treatment of a variety of adult retinal diseases [9]. The ‘free’ form of aflibercept is the pharmacologically active drug moiety, capable of complexing with VEGF. Free aflibercept is cleared by two mechanisms: a fast pathway comprising binding of VEGF to form a VEGF:aflibercept complex and a slower pathway involving other biological mechanisms such as degradation into amino acids. The ‘bound’ aflibercept form in the VEGF:aflibercept complex is incapable of further VEGF binding and is pharmacologically inactive [10, 11]. Systemic pharmacokinetics (PK) and pharmacodynamics (PD) following aflibercept 2 mg per eye have been reported in adult patients with retinal diseases [12]. The aims of this analysis were to describe concentrations of free and adjusted bound aflibercept in plasma of preterm infants with ROP following treatment, investigate the presence of any anti-drug antibodies (ADAs), and explore the relationship between drug exposure and systolic blood pressure (SBP) and diastolic blood pressure (DBP) as markers of systemic anti-VEGF effects, using data from the FIREFLEYE trial [13].

## Patients and methods

FIREFLEYE (NCT04004208) was a 24-week, randomized, open-label, noninferiority trial assessing the efficacy and safety of aflibercept versus laser photocoagulation in infants with treatment-requiring ROP. The protocol (Supplement 1) was reviewed and approved by local ethics committees and institutional review boards at each site before study initiation; written informed consent was obtained before enrollment. An independent data monitoring committee assessed study progress and patient safety. The statistical analysis plan is included in Supplement 2. The study population, randomization process, study procedures and endpoints, and statistical analyses and sample size calculation have been reported in full previously [8].

Infants born at a gestational age of ≤32 weeks or with a birthweight of ≤1500 g who weighed ≥800 g at time of treatment and who had ROP across the entire spectrum of treatment-requiring ROP severities according to the International Classification for ROP [14] (Zone I stage 1+, 2+, 3, 3+; Zone II stage 2+, 3+; or aggressive posterior ROP) in ≥1 eye were randomly assigned 2:1 to receive aflibercept 0.4 mg/eye or laser photocoagulation at baseline. Any additional treatment with aflibercept and laser was prespecified in the protocol. In the aflibercept group, infants could have been retreated with up to 2 additional aflibercept 0.4 mg/eye injections at minimum intervals of 28 days between injections.

The primary outcome was treatment success measured as the proportion of infants without active ROP and unfavorable structural outcomes 24 weeks after starting treatment (investigator-assessed). Secondary endpoints included ocular and systemic treatment-emergent adverse events (TEAE; occurring after the first and not later than 30 days after the last administration of study treatment) and serious adverse events (SAEs) by week 24. Safety assessments included ophthalmic examinations, physical examinations, vital signs, laboratory evaluations, and central nervous system imaging. Blood pressure (BP) was assessed using an automated device appropriate for use in infants, was measured before and after study treatment was administered, and before blood samples were taken.

### Pharmacokinetic, pharmacodynamic, and immunogenicity endpoints

Blood samples for analysis of free and bound aflibercept concentrations were collected from infants in the aflibercept group. Systemic exposure to free aflibercept in plasma (determined by sparse sampling) on day 1 and at weeks 2, 4, 8, 12, and 24 after first dosing and the presence of ADA before and 12 weeks after first dosing were secondary endpoints of the study. The protocol was amended to allow additional sampling beyond week 4 (i.e., at weeks 8, 12, and 24).

### Bioanalytical methods

Assay methods have been previously reported [12]. Validated luminescence-based enzyme-linked immunosorbent assays (ELISAs) were used to determine concentrations of free and bound aflibercept. Both assays were validated in lithium heparin plasma. The lower limits of quantitation (LLOQ) for free and bound aflibercept assays were 15.6 and 31.3 ng/mL, respectively.

The free aflibercept ELISA measures systemic concentrations of aflibercept that is not in complex with VEGF. The bound aflibercept ELISA measures systemic concentrations of aflibercept bound to VEGF forming a VEGF:aflibercept complex. The assay for bound aflibercept was calibrated using the VEGF:aflibercept standards, and results were reported for bound aflibercept as weight per volume of the complex (VEGF:aflibercept). Because 1 ng complex (hVEGF165:aflibercept) equals 0.717 ng aflibercept and 0.283 ng VEGF, the concentration of the complex (bound aflibercept) was multiplied by 0.717 to give an adjusted bound aflibercept concentration.

The presence of anti-aflibercept antibodies (ADA) was assessed in serum samples using a validated titer-based electrochemiluminescence bridging immunoassay. The assay potentially involved three different evaluations of a sample: an initial screen, a confirmation assay, and a titer analysis. The method was developed and validated in accordance with regulatory guidance and industry standards [15, 16]. The sensitivity of the assay, based on the monoclonal antibody positive control, was approximately 7.1 ng/mL.

ADA responses were categorized as low (<1000), medium (1000 to 10,000), and high (>10,000) titers. Positive ADA responses were characterized as either treatment-emergent (negative ADA response at baseline and a positive postdose sample) or treatment-boosted (positive ADA response at baseline and a positive postdose sample >4-fold higher than baseline titer levels).

Samples with a positive ADA response were further tested for presence of neutralizing antibodies (NAbs) using a validated ligand-binding assay. The method indirectly detects the presence of NAbs that prevent the binding of VEGF to aflibercept, with results reported as either positive or negative. The sensitivity of the assay, based on the monoclonal antibody positive control, was approximately 940 ng/mL.

### Statistical analysis

Drug concentration data were derived from infants randomized to the aflibercept group. Variables were analyzed descriptively using frequency tables for categorical variables and sample statistics for continuous variables. Arithmetic mean concentrations were calculated from all individual results, including values below the LLOQ assigned a value of 0. As prespecified (Supplement 2), data were evaluated by baseline weight, gestational age, sex, and race (and, not further detailed in this manuscript, by oxygen supplementation status at baseline, history of sepsis, necrotizing enterocolitis, or intraventricular hemorrhage in a prespecified exploratory analysis).

Graphical assessments of individual and mean plasma drug concentrations versus time were evaluated and summary statistics of the derived parameters generated. Relationships between systemic exposure and specific safety parameters, including BP and treatment-emergent serious adverse events (TESAE), were explored graphically. For each sampling time point, scatterplots for individual aflibercept concentrations (free and adjusted bound) versus the safety marker (SBP, DBP, change from baseline in SBP and DBP) were created. Infants with unilateral and bilateral treatment are presented separately, as well as infants with TESAEs within 30 days of the first aflibercept injection and those without.

## Results

**Fig. 1:**
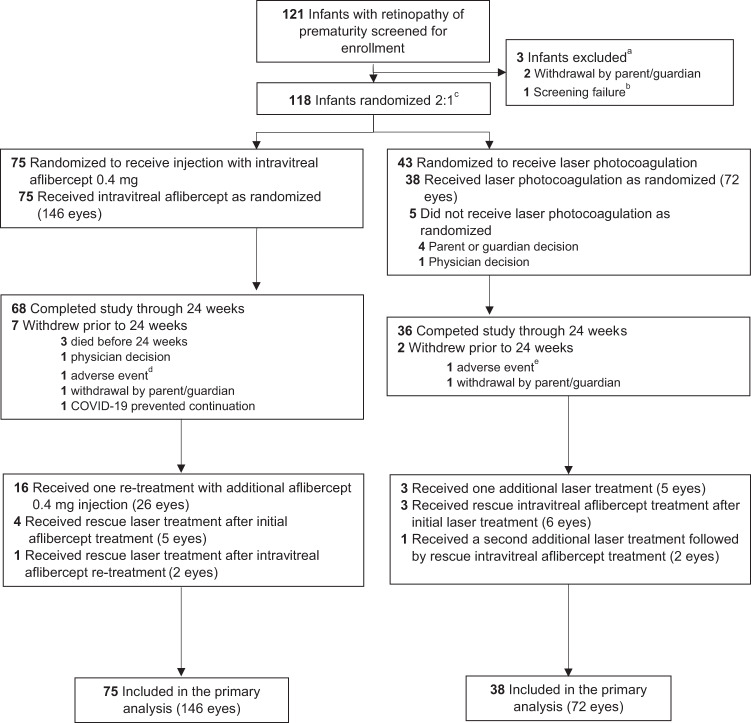
Screening, randomization, and follow-up in the FIREFLEYE trial. ^a^See eMethods for additional details of inclusion and exclusion criteria. ^b^One infant with retinopathy of prematurity only in zone III was screened but not randomized. ^c^Randomization was stratified by retinopathy of prematurity category (zones) and country of enrollment. Randomization and evaluation were by infant, with each infant demonstrating retinopathy in 1 eye or both eyes. ^d^One infant was discontinued from the trial after an adverse event of sinus tachycardia. ^e^One infant was discontinued from the trial after an adverse event of retinal detachment.

Of 113 treated infants, 68 of 75 (90.7%) in the aflibercept group and 36 of 38 (94.7%) in the laser group completed the study (Fig. 1). Baseline demographics and characteristics are listed in Supplementary eTable. 1, and have been previously reported along with efficacy and safety results [8]. Mean bodyweight increased with chronological age (Supplementary eFig. 1) and was numerically slightly higher in the aflibercept group compared with the laser group at baseline (2.022 vs. 1.851 kg) and week 24 (6.148 vs. 5.765 kg).

### Pharmacokinetics

Aflibercept treatment at baseline was administered at a mean (SD) chronological age of 10.4 (2.8) weeks and was mostly bilateral (71 of 75 infants [94.7%]). Of 146 eligible eyes, 26 (17.8%) received 1 retreatment and no infant received more than 2 injections (1 retreatment) per eye.

**Fig. 2:**
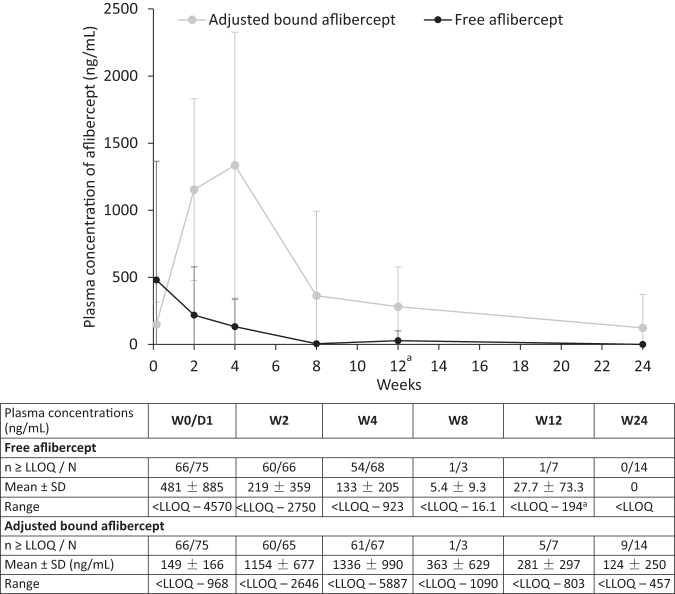
Arithmetic mean ± SD concentrations of free and adjusted bound aflibercept (ng/mL) in plasma (in bilaterally and unilaterally treated infants combined). *D* day, *LLOQ* lower limit of quantitation, *VEGF* vascular endothelial growth factor, *W* week. ^a^A detectable concentration of free aflibercept was measured at week 12 (194 ng/mL) in 1 infant who received aflibercept retreatment in both eyes at week 11. Values below the LLOQ were substituted by 0 for the calculation of statistics. LLOQ was 15.6 ng/mL for free aflibercept and 31.3 ng/mL for bound aflibercept. The concentration of the bound aflibercept complex was adjusted by multiplying by 0.717 to account for the VEGF present in the bound complex (adjusted bound aflibercept).

Concentrations of free aflibercept in plasma were highly variable between infants. Maximum concentration (C_max_) was measured at day 1 and declined thereafter, with mean (SD) concentrations overall in bilaterally and unilaterally treated infants of 481 (885) ng/mL on day 1 and 133 (205) ng/mL at week 4 (Fig. 2). Individual concentration values for free aflibercept in plasma were almost all below the LLOQ of the assay after 8 weeks and onwards. At week 8, only 1 of 3 infants had a detectable free aflibercept concentration (16.1 ng/mL, close to the LLOQ). Of 7 infants with a sample collected at week 12, a detectable free aflibercept concentration (194 ng/mL) was measured in only 1 infant who had received aflibercept retreatment in both eyes at week 11. Free aflibercept concentrations were undetectable in all patients at week 24.

Concentrations of adjusted bound aflibercept in plasma increased from day 1 to week 4 in bilaterally and unilaterally treated infants and decreased thereafter through week 24. In the 4 infants treated unilaterally, mean concentrations of adjusted bound aflibercept at weeks 2 and 4 were approximately half of those in bilaterally treated infants (Supplementary eFig. 2).

At each time point, mean free and adjusted bound aflibercept concentrations in plasma were highest in the lowest bodyweight group and lowest in the highest bodyweight group. In all bodyweight groups, mean free aflibercept concentrations declined from day 1 onward and mean adjusted bound aflibercept concentrations increased from day 1 until week 4 and declined thereafter (Supplementary eFig. 3). There were generally no observed differences in exposure to free aflibercept between gestational age groups (Supplementary eFig. 4) or subgroups based on sex, race, oxygen supplementation at baseline, history of sepsis, necrotizing enterocolitis, or intraventricular hemorrhage (data not shown).

### Pharmacodynamics

**Fig. 3:**
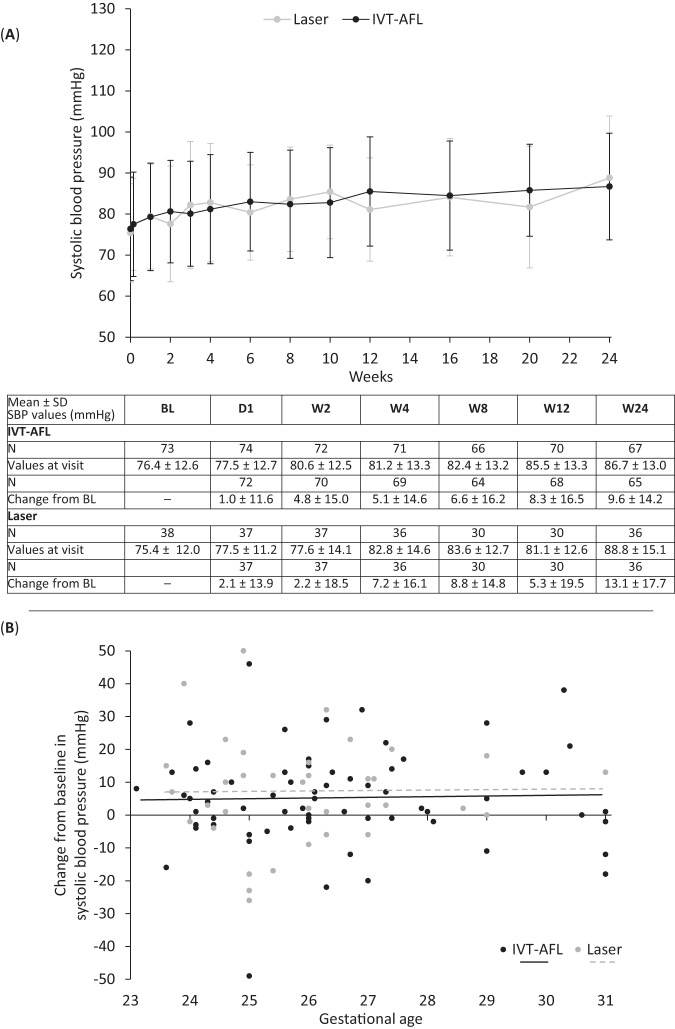
Systolic blood pressure. **A** Arithmetic mean ± SD systolic blood pressure through week 24 in infants with retinopathy of prematurity in both treatment groups (aflibercept vs. laser photocoagulation) and **B** mean change from baseline in systolic blood pressure at week 4 after baseline treatment according to gestational age at birth. *BL* baseline, *D* day, *N* number of observations, *SBP* systolic blood pressure, *W* week. In panel **A**, *n* = 73 (baseline), *n* = 74 (day 1), *n* = 72 (week 1), *n* = 72 (week 2), *n* = 71 (week 3), *n* = 71 (week 4), *n* = 71 (week 6), *n* = 66 (week 8), *n* = 66 (week 10), *n* = 70 (week 12), *n* = 69 (week 16), *n* = 63 (week 20), *n* = 67 (week 24) for aflibercept and *n* = 38 (baseline), *n* = 37 (day 1), *n* = 37 (week 1), *n* = 37 (week 2), *n* = 37 (week 3), *n* = 36 (week 4), *n* = 34 (week 6), *n* = 30 (week 8), *n* = 30 (week 10), *n* = 30 (week 12), *n* = 34 (week 16), *n* = 30 (week 20), *n* = 36 (week 24) for laser. Data are not shown for infants whose BP was taken outside scheduled study visits. Week 1, day 1: *n* = 2 (aflibercept) and *n* = 2 (laser); week 2, day 1: *n* = 1 (aflibercept); week 3, day 1: *n* = 1 (laser); week 4, day 1: *n* = 2 (aflibercept); week 5, *n* = 4 (aflibercept) and *n* = 2 (laser); week 5, day 1: *n* = 1 (aflibercept) and *n* = 2 (laser); week 6, day 1: *n* = 1 (aflibercept) and *n* = 2 (laser); week 7: *n* = 2 (aflibercept) and *n* = 1 (laser); week 8, day 1, *n* = 1 (aflibercept); week 9: *n* = 1 (aflibercept) and *n* = 1 (laser); week 10, day 1: *n* = 3 (aflibercept) and *n* = 1 (laser); week 11: *n* = 5 (aflibercept); week 11, day 1: *n* = 3 (aflibercept); week 12, day 1: *n* = 3 (aflibercept); week 13: *n* = 2 (aflibercept); week 14: *n* = 3 (aflibercept); week 14, day 1: *n* = 1 (aflibercept); week 15: *n* = 2 (aflibercept); week 15, day 1: *n* = 1 (aflibercept); week 16, day 1: *n* = 4 (aflibercept); week 17: *n* = 4 (aflibercept); week 18: *n* = 1 (aflibercept). All data are shown in Supplementary eFig. 5. In panel **B**, individual observations in the aflibercept and laser groups are shown by black and gray circles, respectively. The dotted line depicts the regression line for laser; the solid line, the regression line for aflibercept.

**Fig. 4:**
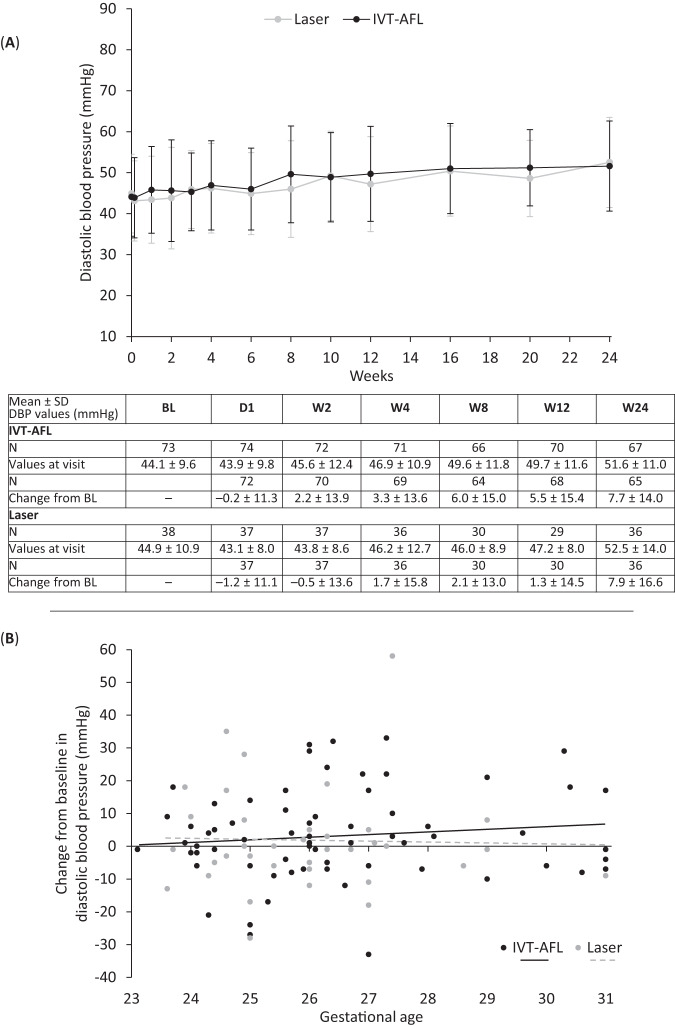
Diastolic blood pressure. **A** Arithmetic mean ± SD diastolic blood pressure through week 24 in infants with retinopathy of prematurity in both treatment groups (aflibercept vs. laser photocoagulation) and **B** mean change from baseline in diastolic blood pressure at week 4 after baseline treatment according to gestational age at birth. *BL* baseline, *D* day, *N* number of observations, *SBP* systolic blood pressure, *SD* standard deviation, *W* week. In panel **A**, *n* = 73 (baseline), *n* = 74 (day 1), *n* = 72 (week 1), *n* = 72 (week 2), *n* = 71 (week 3), *n* = 71 (week 4), *n* = 71 (week 6), *n* = 66 (week 8), *n* = 66 (week 10), *n* = 70 (week 12), *n* = 69 (week 16), *n* = 63 (week 20), *n* = 67 (week 24) for aflibercept and *n* = 38 (baseline), *n* = 37 (day 1), *n* = 37 (week 1), *n* = 37 (week 2), *n* = 37 (week 3), *n* = 36 (week 4), *n* = 34 (week 6), *n* = 30 (week 8), *n* = 30 (week 10), *n* = 30 (week 12), *n* = 34 (week 16), *n* = 30 (week 20), *n* = 36 (week 24) for laser. Data are not shown for infants whose BP was taken outside scheduled study visits. Week 1, day 1: *n* = 2 (aflibercept) and *n* = 2 (laser); week 2, day 1: *n* = 1 (aflibercept); week 3, day 1: *n* = 1 (laser); week 4, day 1: *n* = 2 (aflibercept); week 5, *n* = 4 (aflibercept) and *n* = 2 (laser); week 5, day 1: *n* = 1 (aflibercept) and *n* = 2 (laser); week 6, day 1: *n* = 1 (aflibercept) and *n* = 2 (laser); week 7: *n* = 2 (aflibercept) and *n* = 1 (laser); week 8, day 1: *n* = 1 (aflibercept); week 9: *n* = 1 (aflibercept) and *n* = 1 (laser); week 10, day 1: *n* = 3 (aflibercept) and *n* = 1 (laser); week 11: *n* = 5 (aflibercept); week 11, day 1: *n* = 3 (aflibercept); week 12, day 1: *n* = 3 (aflibercept); week 13: *n* = 2 (aflibercept); week 14: *n* = 3 (aflibercept); week 14, day 1: *n* = 1 (aflibercept); week 15: *n* = 2 (aflibercept); week 15, day 1: *n* = 1 (aflibercept); week 16, day 1: *n* = 4 (aflibercept); week 17: *n* = 4 (aflibercept); week 18: *n* = 1 (aflibercept). All data are shown in Supplementary eFig. 5. In panel **B**, individual observations in the aflibercept and laser groups are shown by black and gray circles, respectively. The dotted line depicts the regression line for laser and the solid line, the regression line for aflibercept.

BP courses were similar in both treatment groups. Mean SBP values (76.4 and 75.4 mm Hg in the aflibercept and laser groups, respectively, at baseline) steadily increased in both groups to 86.7 and 88.8 mm Hg at week 24 (Fig. 3A and Supplementary eFig. 5), in line with increases in bodyweight and maturation of BP regulation. Mean DBP values were also similar in the aflibercept and laser groups (increasing from 44.1 and 44.9 mm Hg at baseline to 51.6 and 52.5 mm Hg at week 24, respectively; Fig. 4A and Supplementary eFig. 5). Over the ranges studied, there was no correlation between gestational age and changes from baseline to week 4 in SBP (Fig. 3B) and DBP (Fig. 4B) in either treatment group, nor between bodyweight and changes in SBP or DBP (Supplementary eFig. 6).

**Fig. 5:**
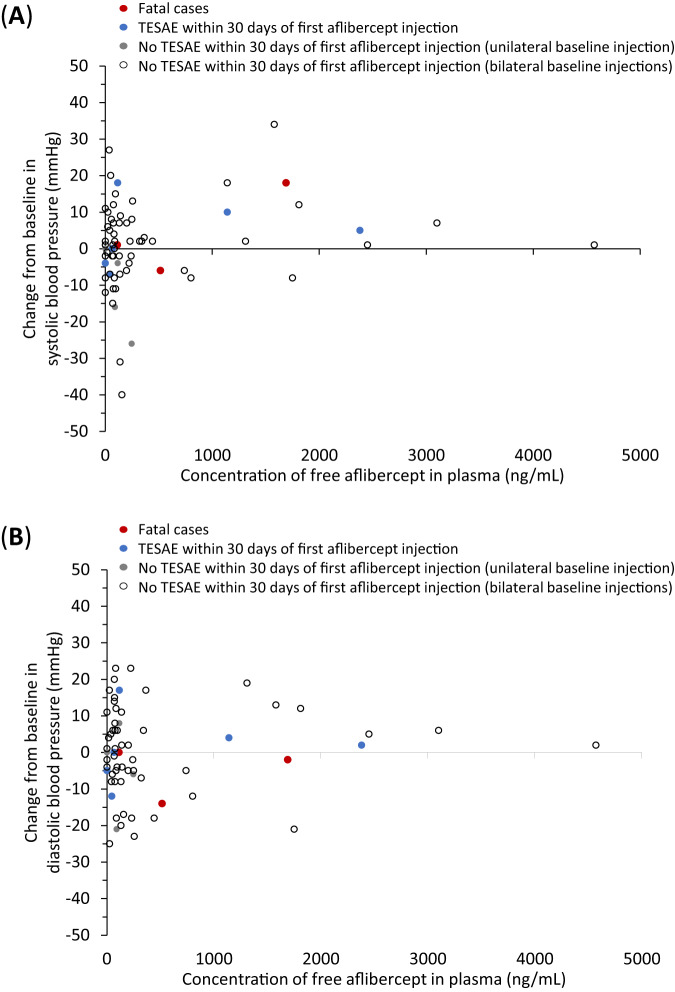
Blood pressure versus concentrations of free aflibercept in plasma. Change from baseline to day 1 in **A** systolic blood pressure and **B** diastolic blood pressure versus concentrations of free aflibercept in plasma at day 1 for individual infants. *BP* blood pressure, *LLOQ* lower limit of quantitation, *TESAE* treatment-emergent serious adverse event. Infants with TESAEs during the first 30 days following the start of treatment who had missing values for either aflibercept plasma concentrations and/or BP values at single time points are not included in the figure. Values below LLOQ were substituted by 0. LLOQ was 15.6 ng/mL for free aflibercept.

There was no association between concentrations of free aflibercept and change in SBP or DBP (shown for day 1 and week 2 in Fig. 5 and Supplementary eFig. 7, respectively). There was also no association between concentrations of adjusted bound aflibercept and changes in SBP and DBP (shown for week 4 in Supplementary eFig. 8).

### Adverse events

Adverse events (AEs) have been described in detail [8] and are compatible with underlying clinical events/conditions of preterm infants. There was no indication infants with higher individual free aflibercept concentrations had clusters of AEs due to systemic effects. Of 9 infants who experienced TESAEs, these events occurred within 30 days of the first aflibercept injection in 6 cases (1 case each of pneumonia aspiration; retinal detachment; COVID-19 and pneumonia; worsening of ROP; increased intraocular pressure; corneal edema and overdose; bronchiolitis; Supplementary eTable 2). Datapoints from these infants were distributed across the entire range of concentrations of free aflibercept concentrations and adjusted bound aflibercept (Fig. 5 and Supplementary eFigs. 7, 8). In 3 infants who died (57‒144 days after the first aflibercept injection), free and adjusted bound concentrations were also within the observed range of the aflibercept group (Supplementary eTable 3). One patient in each treatment group had a TEAE of transient, mild, asymptomatic proteinuria. No AE/SAE of hypertension was reported throughout the study.

### Immunogenicity

Overall, the incidence of treatment-emergent ADA response in the aflibercept group was low. One infant demonstrated a treatment-emergent ADA response, with low-titer (1:30) and no NAb. This low level of immunogenicity had no effect on drug exposure, efficacy, or safety. The concentration of free aflibercept in this infant was within the range observed in the overall population at weeks 2 and 4 (28.3 ng/mL and 28.6 ng/mL respectively). No safety concerns were identified and this infant responded to aflibercept treatment.

## Discussion

In preterm infants with ROP treated with aflibercept 0.4 mg/eye, mean systemic concentrations of free aflibercept declined from day 1 to 28 and individual concentrations of free aflibercept were almost all below the LLOQ within 8 weeks. Mean concentrations of adjusted bound aflibercept reached their maximum 4 weeks after dosing and declined thereafter.

Notably, concentrations of aflibercept were highly variable and group sizes were small. Analyses revealed no clinically relevant differences regarding free or adjusted bound aflibercept concentrations in plasma in subpopulations by sex, race, or gestational age. Mean free and adjusted bound aflibercept concentrations were highest in the lowest bodyweight group and vice versa. However, lower bodyweight at baseline treatment appeared not to be predictive of occurrence and severity of AEs. Concentrations of free and adjusted bound aflibercept in infants with TESAEs were distributed across the entire concentration range at all measured time points and no clinically relevant ADA development occurred.

Aflibercept treatment of ROP is short term (in FIREFLEYE, maximum 2 injections per eye [8]) compared to the long-term treatment of adult retinal diseases. Following intravitreal injection of a 2 mg dose in adults, aflibercept is released from the eye into the systemic circulation, where it is predominately observed as bound, inactive aflibercept in a stable complex with VEGF [12, 17]. In an adult PK substudy, the mean free aflibercept C_max_ in plasma was approximately 20 ng/mL (range, 0–54 ng/mL) attained within 1‒3 days after a 2 mg intravitreal injection; concentrations were undetectable 2 weeks postdose in almost all patients [12]. In this study of infants with ROP treated with aflibercept 0.4 mg/eye, the mean C_max_ of free aflibercept was higher than in adult patients after aflibercept 2 mg administration (by a factor of approximately 24), and the C_max_ of bound aflibercept was measured later (at 4 weeks instead of 7 days as in adults). In the RAINBOW study of ranibizumab, median C_max_ was also higher in infants compared with adults (11.5–24.3 vs. 1.5 ng/mL) [18].

As a protein-based therapeutic, elimination of aflibercept is known to be dependent on specific, target-mediated processes and unspecific, nontarget-mediated routes of proteolysis [19, 20]. Both processes apply to free aflibercept, whereas bound aflibercept is eliminated by proteolysis only. Basal metabolic rates of preterm infants have been described as lower than those of full-term infants, increasing at slower rates during the first month of extrauterine life [21]. Thus, it may be speculated that proteolytic processes are also still developing in preterm infants, resulting in slower nontargeted elimination processes, which may contribute to the higher drug concentrations and later time to peaks observed in preterm neonates compared with the adult population.

In addition, the Fc domain of IgG1 is involved in the distribution and elimination processes of aflibercept through its interaction with the neonatal Fc receptor (FcRn) [22]. The expression of FcRn has recently been noted to be dependent on gestational age, being highest during the third trimester [23]. As the ligand-binding portion of the VEGF receptor is fused to the Fc domain, it is hypothesized that factors related to Fc receptor kinetics in different tissues may impact the distribution and elimination of free and bound aflibercept. Characteristics of aflibercept have previously been described in a number of studies [17, 24–26], and data from aflibercept studies in adult patients indicate the systemic half-life of free aflibercept is shorter compared to typical Fc-containing antibodies and apparent systemic clearance may be dominated by target-mediated and absorption processes [10, 12, 27, 28]. However, this may be different in preterm infants as age dependencies of relevant processes are not well characterized [29]. As an example, endogenous IgG levels are age-dependent and serum concentrations decrease by approximately 50% during the first months of extrauterine life. Competition between endogenous and exogenous Fc-containing proteins for receptor binding has been identified as an important factor in the disposition of IgG-containing therapeutics [30] and may help explain why aflibercept is cleared differently in adults and infants.

VEGF levels in plasma were not measured in this trial since Sumner et al. have reported that VEGF inhibitors such as aflibercept, ranibizumab, and bevacizumab interfere with quantification of free VEGF in the Quantikine Human VEGF ELISA in proportion to their relative binding affinity for VEGF, and free VEGF concentrations may be overestimated for VEGF inhibitors that bind VEGF in a 2:1 stoichiometry (ranibizumab, bevacizumab) compared with aflibercept, which binds VEGF in a 1:1 stoichiometry [31]. These authors also reported marked differences of circulating VEGF concentrations for studies where aflibercept was administered intravitreally and different bioanalytical assays were used to quantify free VEGF. The effect of 0.4 mg/eye intravitreal administration in pediatric patients with ROP on systemic VEGF levels can be indirectly deduced from adjusted bound aflibercept concentrations in plasma, as they reflect binding of free aflibercept to systemic endogenous VEGF. In healthy adults, saturation of binding to systemic VEGF occurs only at high (≥2 mg/kg) intravenous doses [32], with mean free and adjusted bound aflibercept C_max_ values of 38,600 ng/mL and 2,380 ng/mL, respectively [33]. Mean free aflibercept and adjusted bound C_max_ in pediatric patients with ROP after 0.4 mg/eye intravitreal administration are approximately 80 times and 1.8 times lower, respectively, than that for the 2 mg/kg intravenous dose in adults, while baseline systemic VEGF concentrations are much higher in patients with ROP than healthy adults [34–37].

BP variability was high in our study, although mean SBP and DBP showed the expected development over time, with increasing chronological age and bodyweight having the expected positive correlation with BP [38]. There was no relevant difference between the treatment groups over the 24-week study duration, and mean BP values were within expected ranges for preterm infants [39, 40]. Importantly, the data provide no evidence suggestive of a causal association between aflibercept treatment and development of arterial hypertension or proteinuria.

In summary, this was the first randomized prospective collection of PK, PD, and immunogenicity data in this vulnerable pediatric population of preterm infants with ROP treated with aflibercept. We should acknowledge limitations of this study: the relatively small sample size, which is consistent with the rarity of the condition under investigation [41]; scarcity of data collected beyond week 8; and the overall follow-up span of 24 weeks.

We can, however, conclude from currently available data that the use of aflibercept 0.4 mg/eye in preterm infants with acute ROP was not associated with clinically observable systemic effects on BP or associated TEAEs up to 24 weeks post-injection. The clinically apparent AE profile was consistent with the established profile of intravitreal aflibercept 2 mg in adults, and long-term follow-up of infants is continuing in the phase 3b extension study FIREFLEYE Next. This ongoing follow-up study will deliver data on ocular and further clinical outcomes, including growth and neurodevelopmental outcomes, through 5 years of age following treatment of acute-phase ROP in FIREFLEYE with intravitreal aflibercept 0.4 mg vs. laser photocoagulation.

Intravitreal aflibercept has been approved for treatment of acute ROP in Japan (September 2022), the European Union (December 2022), Switzerland, Great Britain, the USA (February 2023), and Brazil (April 2023) [9, 42–44].

## Summary

### What was known before

- Systemic pharmacokinetics (PK) and pharmacodynamics (PD) following intravitreal aflibercept have been reported for adult patients with retinal diseases
- Intravitreal aflibercept is also approved for the treatment of acute-phase retinopathy of prematurity (ROP) in preterm neonates

### What this study adds

- Following intravitreal aflibercept (0.4 mg per eye) for treatment of ROP, concentrations of free and bound aflibercept are not causally associated with clinically relevant effects of blood pressure or adverse events up to week 24
- Long-term follow-up to 5-years of age, assessing ocular and further clinical outcomes is ongoing

### Supplementary information

Supplementary Material

## Data Availability

Availability of the data underlying this publication will be determined later according to Bayer’s commitment to the EFPIA/PhRMA “Principles for responsible clinical trial data sharing.” This pertains to scope, time point and process of data access. As such, Bayer commits to sharing upon request from qualified scientific and medical researchers patient-level clinical trial data, study-level clinical trial data, and protocols from clinical trials in patients for medicines and indications approved in the United States (US) and European Union (EU) as necessary for conducting legitimate research. This applies to data on new medicines and indications that have been approved by the EU and US regulatory agencies on or after January 01, 2014. Interested researchers can use www.clinicalstudydatarequest.com to request access to anonymized patient-level data and supporting documents from clinical studies to conduct further research that can help advance medical science or improve patient care. Information on the Bayer criteria for listing studies and other relevant information is provided in the study sponsors section of the portal. Data access will be granted to anonymized patient-level data, protocols, and clinical study reports after approval by an independent scientific review panel. Bayer is not involved in the decisions made by the independent review panel. Bayer will take all necessary measures to ensure that patient privacy is safeguarded.
