# Correction: Systemic exposure to aflibercept after intravitreal injection in premature neonates with retinopathy of prematurity: results from the FIREFLEYE randomized phase 3 study

**DOI:** 10.1038/s41433-024-02948-y

**Published:** 2024-02-08

**Authors:** Andreas Stahl, Noriyuki Azuma, Wei-Chi Wu, Domenico Lepore, Emine Sukgen, Hidehiko Nakanishi, Jan Mazela, Sergio Leal, Alexander Pieper, Sarah Schlief, Thomas Eissing, Kenneth C. Turner, An Zhao, Julia Winkler, Joachim Höchel, Evra Köfüncü, Torsten Zimmermann, on behalf of the FIREFLEYE Study Group

**Affiliations:** 1 Department of Ophthalmology, University Medicine Greifswald, Greifswald, Germany; 2 Department of Ophthalmology and Laboratory for Visual Science, National Centre for Child Health and Development, Tokyo, Japan; 3 Department of Developmental and Regenerative Biology, Medical Research Institute, Tokyo Medical and Dental University, Tokyo, Japan; 4 Department of Ophthalmology, Linkou Chang Gung Memorial Hospital, and College of Medicine, Chang Gung University, Taoyuan, Taiwan; 5 Department of Geriatrics and Neuroscience, Catholic University of the Sacred Heart, A. Gemelli Foundation IRCCS, Rome, Italy; 6 Department of Ophthalmology, Health Science University, Adana City Training and Research Hospital, Adana, Turkey; 7 Research and Development Center for New Medical Frontiers, Department of Advanced Medicine, Division of Neonatal Intensive Care Medicine, Kitasato University School of Medicine, Sagamihara, Japan; 8 Department of Neonatology, Poznan University of Medical Sciences, Poznan, Poland; 9 Bayer Consumer Care AG, Basel, Switzerland; 10 Chrestos Concept GmbH & Co., Essen, Germany; 11 Bayer AG, Berlin, Germany; 12 Bayer AG, Leverkusen, Germany; 13 Regeneron Pharmaceuticals Inc., Tarrytown, NY USA; 14 OCCAMS, Amstelveen, the Netherlands

**Keywords:** Retinal diseases, Outcomes research, Paediatrics, Adverse effects

Correction to: *Eye* 10.1038/s41433-023-02919-9, published online 10 January 2024

In the original article, the name of the author Joachim Höchel was incorrectly given as ‘Joachim Hoechel’. In addition, several corrections have been made in the ‘Discussion’ section of the article.

The following text section in the first paragraph was removed:

“VEGF levels in plasma were not measured in this trial but can be indirectly inferred from the reported concentrations of free afibercept: providing signifcant concentrations of free afibercept are observed (up to week 4), there are very likely no free VEGF molecules measurable in the systemic circulation (otherwise they would bind to afibercept) [17]. Inversely, by the time free afibercept can no longer be measured in the circulation (by week 8), free systemic plasma VEGF levels are likely to have increased.”

The sentence “Analyses revealed no clinically relevant differences regarding free or adjusted bound aflibercept concentrations in plasma in subpopulations by sex, race, and gestational age” was corrected to read as follows: “Analyses revealed no clinically relevant differences regarding free or adjusted bound aflibercept concentrations in plasma in subpopulations by sex, race, or gestational age”.

Furthermore, the caption for Figure 5B, has been corrected from “diastolic blood pressure versus concentrations of free aflibercept in plasma at day 1” to “diastolic blood pressure versus concentrations of free aflibercept in plasma at day 1 for individual infants."

The following paragraph has been added as paragraph six in the ‘Discussion’ section of the article:

“VEGF levels in plasma were not measured in this trial since Sumner et al. have reported that VEGF inhibitors such as aflibercept, ranibizumab, and bevacizumab interfere with quantification of free VEGF in the Quantikine Human VEGF ELISA in proportion to their relative binding affinity for VEGF, and free VEGF concentrations may be overestimated for VEGF inhibitors that bind VEGF in a 2:1 stoichiometry (ranibizumab, bevacizumab) compared with aflibercept, which binds VEGF in a 1:1 stoichiometry [31]. These authors also reported marked differences of circulating VEGF concentrations for studies where aflibercept was administered intravitreally and different bioanalytical assays were used to quantify free VEGF. The effect of 0.4 mg/eye intravitreal administration in pediatric patients with ROP on systemic VEGF levels can be indirectly deduced from adjusted bound aflibercept concentrations in plasma, as they reflect binding of free aflibercept to systemic endogenous VEGF. In healthy adults, saturation of binding to systemic VEGF occurs only at high (≥2 mg/kg) intravenous doses [32], with mean free and adjusted bound aflibercept C_max_ values of 38,600 ng/mL and 2380 ng/mL, respectively [33]. Mean free aflibercept and adjusted bound C_max_ in pediatric patients with ROP after 0.4 mg/eye intravitreal administration are approximately 80 times and 1.8 times lower, respectively, than that for the 2 mg/kg intravenous dose in adults, while baseline systemic VEGF concentrations are much higher in patients with ROP than healthy adults [34–37].”

Finally, reference 17 (Sumner G, Georgaros C, Rafique A, DiCioccio T, Martin J, Papadopoulos N, et al. Anti-VEGF drug interference with VEGF quantitation in the R&D systems human quantikine VEGF ELISA kit. Bioanalysis. 2019;11:381–92) has been moved from place 17 to place 31 in the list. References 32–37 have been added to the list:

32. Thai HT, Veyrat-Follet C, Vivier N, Dubruc C, Sanderink G, Mentre F et al. A mechanism-based model for the population pharmacokinetics of free and bound aflibercept in healthy subjects. Br J Clin Pharmacol. 2011;72:402–14.

33. U.S. Food and Drug Administration, Center for Drug Evaluation and Research. Zaltrap Original BLA, 125418Orig1s000. February 2012. Available at: https://www.accessdata.fda.gov/drugsatfda_docs/nda/2012/125418Orig1s000ClinPharmR.pdf Last accessed November 2023.

34. Ahuja S, Saxena S, Akduman L, Meyer CH, Kruzliak P, Khanna VK. Serum vascular endothelial growth factor is a biomolecular biomarker of severity of diabetic retinopathy. Int J Retina Vitreous. 2019;5:29.

35. Wang J, Chen S, Jiang F, You C, Mao C, Yu J et al. Vitreous and plasma VEGF levels as predictive factors in the progression of proliferative diabetic retinopathy after vitrectomy. PLoS One. 2014;9:e110531.

36. Kut C, Mac Gabhann F, Popel AS. Where is VEGF in the body? A meta-analysis of VEGF distribution in cancer. Br J Cancer. 2007;97:978–85.

37. Huang CY, Lien R, Wang NK, Chao AN, Chen KJ, Chen TL et al. Changes in systemic vascular endothelial growth factor levels after intravitreal injection of aflibercept in infants with retinopathy of prematurity. Graefes Arch Clin Exp Ophthalmol. 2018;256:479–87.

The numbering of the references within the text has also been adjusted accordingly.

The original article has been corrected.

