# Supplementary material for: Systemic exposure to aflibercept after intravitreal injection in premature neonates with retinopathy of prematurity: results from the FIREFLEYE randomized phase 3 study
Source: Eye (Lond). 2024 Jan 10;38(8):1444–53. doi: 10.1038/s41433-023-02919-9 (PMC11126565; doi:10.1038/s41433-023-02919-9)
Supplement: Supplementary file 1 — Supplementary Material [file 41433_2023_2919_MOESM1_ESM.pdf]

Systemic exposure to aflibercept after intravitreal injection in premature neonates with retinopathy of prematurity: results from the FIREFLEYE randomized phase 3 study

Supplementary Materials

FIREFLEYE enrolling sites and principal investigators

Argentina: Pablo Larrea (Hospital Público Descentralizado "Dr. Guillermo Rawson", San Juan)
Austria: Matthias Bolz (Kepler Universitätsklinikum Campus III, Linz)
Belgium: Patricia Delbeke (AZ St-Jan Brugge Oostende AV, Brugge)
Brazil: Nilva de Moraes (UNIFESP/EPM, Sao Paulo); Maria Regina Bentlin (Hospital das Clínicas de Botucatu - UNESP Botucatu, Botucatu)
Bulgaria: Violeta Chernodrinska (II SOGHAT Sheinovo, Sofia); Christina Grupcheva (SHOGAT Prof Dimitar Stamatov, Varna); Liliyana Dimitrova (Acibadem City Clinic Multiprofile Hospital for Active Treatment, Sofia); Vasil Marinov (UMHAT Sveti Georgi, Plovdiv)
Czech Republic: Magdalena Kovacova (Vseobecna fakultni nemocnice v Praze, Praha); Juraj Timkovic (Fakultni nemocnice Ostrava, Ostrava)
Greece: Ioannis Asproudis (University General Hospital of Ioannina, Ioannina); Agathi Kouri (P & A KYRIAKOU Children's Hospital, Athens); Asimina Mataftsi (Papageorgiou General Hospital of Thessaloniki, Thessaloniki)
Hong Kong: Wai Ching Lam (Queen Mary Hospital, Hong Kong)
Hungary: Erzsebet Princzkel (EKBC, Uj Szent Janos Korhaz es Szakrendelo, Budapest)
Israel: Hana Leiba (Kaplan Medical Center, Rehovot)
Italy: Luca Buzzonetti (IRCCS Ospedale Pediatrico Bambino Gesù, Rome); Carlo Cagini (Ospedale S. Maria della Misericordia, Perugia); Domenico Lepore (Catholic University of the Sacred Heart, A. Gemelli Foundation IRCSS, Rome): Silvia Gabriella Osnaghi (Fondazione IRCCS Ca' Granda Ospedale Maggiore Policlinico, Milan)
Japan: Mitsuru Arima (Kyushu University Hospital, Fukuoka); Hideyuki Hayashi (Fukuoka University Hospital, Fukuoka); Tomoka Kambe (Saitama Children's Medical Center, Saitama); Mariko Kiyota (Tokyo Metropolitan Bokutoh Hospital, Sumida-ku); Hiroyuki Kondo (University of Occupational and Environmental Health, Kitakyushu); Shunji Kusaka (Kindai University Hospital, Osakasayama); Tomoko Miyazato (Okinawa Prefectural Nanbu Medical Center and Children’s Medical Center, Shimajiri-gun); Eiichiro Noda (Tokyo Metropolitan Children's Medical Center, Fuchu); Yuta Saito (Showa University Hospital, Shinagawa); Yasunobu Saneyoshi (Kurume University Hospital, Kurume); Tetsuju Sekiryu (Fukushima Medical University Hospital, Fukushima); Takako Tachikawa (Tokyo Metropolitan Ohtsuka Hospital, Toshima-ku)
Malaysia: Nor Akmal Bahari (Hospital Kuala Lumpur, Kuala Lumpur)

The Netherlands: Stefan de Geus (Maxima Medisch Centrum, Veldhoven)

Poland: Jan Mazela (Poznan University of Medical Sciences, Poznan)

Portugal: Ana Almeida (CHLO - Hospital Sao Francisco Xavier, Lisbon); Susana Teixeira (Hospital Prof. Dr Fernando Fonseca, Amadora)

Romania: Narcis Berlea (Spitalul Clinic de Obstretica si Ginecologie "Cuza Voda", Iasi); Delia Nicoara (Clinical Emergency County Hospital, Cluj-Napoca)

Russian Federation: Vladimir Brzheskiy (Pediatric Medical University, Saint-Petersburg); Yulia Gorelik (City Children Hospital №1, Saint-Petersburg); Eyvgeny Sidorenko (Russian National Scientific Medical University, Moscow); Eyvgeny Sidorenko (FGBUZ "NPC of special children care n.a. Voino-Yaseneckogo", Moscow); Irina Trifanenkova (FSAI NMRC IRTC "Eye Microsurgery", Kaluga)

Singapore: Gavin Tan (KK Women's and Children's Hospital, Singapore)

Slovakia: Dana Tomcikova (Narodny ustav detskych chorob, Bratislava)

South Korea: Sang Jin Kim (Samsung Medical Center, Seoul); So Young Kim (Soon Chun Hyang University Cheonan Hospital, Cheonan-si); Joo Yong Lee (Asan Medical Center, Seoul)

Spain: Jesús Peralta Calvo (Hospital Universitario "La Paz", Madrid); Julia Escudero Gómez (Hospital Regional de Málaga, Málaga); Pilar Tejada Palacios (Hospital Universitario 12 de Octubre, Madrid)

Sweden: Ann Hellström (Sahlgrenska Universitetssjukhuset, Göteborg)

Taiwan: Yu-Hung Lai (Kaohsiung Medical University Chung-Ho Memorial Hospital, Kaohsiung); Hsiang-Ling Tsai (Mackay Memorial Hospital, Taipei)

Turkey: Imren Vardarli Akkoyun (Baskent Universitesi Tip Fakultesi Hastanesi, Ankara); Hikmet Basmak (Eskisehir Osmangazi Universitesi Tip Fakultesi, Eskisehir); Sibel Caliskan Kadayifcilar (Hacettepe Universitesi Tip Fakultesi, Ankara); Muhammet Erol (Saglik Bilimleri Universitesi Antalya EA Hastanesi, Antalya); Sengul Ozdek (Gazi Universitesi Tip Fakultesi, Ankara); Emine Sukgen (Saglik Bilimleri Universitesi, Adana)

UK: Sally Painter (Birmingham Women’s Hospital, Birmingham)

Ukraine: Mykola Aryayev (Odesa Regional Children's Clinical Hospital, Odesa)

Supplementary Figures and Tables

Supplementary eTable 1. Baseline demographics and characteristics of infants randomized to receive intravitreal aflibercept 0.4 mg in FIREFLEYE

Supplementary eFig. 1. Arithmetic mean ± SD body weight through week 24 in infants with retinopathy of prematurity in both treatment groups (aflibercept vs. laser photocoagulation).

Supplementary eFig. 2. Arithmetic mean ± SD concentrations of free and adjusted bound aflibercept (ng/mL) in plasma from unilaterally or bilaterally treated infants.

Supplementary eFig. 3. Arithmetic mean ± SD plasma concentrations of (**a**) free and (**b**) adjusted bound aflibercept (ng/mL) in plasma, by body weight at baseline treatment.

Supplementary eFig. 4. Arithmetic mean ± SD plasma concentrations of (**a**) free and (**b**) adjusted bound aflibercept (ng/ml) in plasma, by gestational age at baseline treatment.

Supplementary eFig. 5. Arithmetic mean ± SD systolic and diastolic blood pressure through week 24 (including visits outside those scheduled in the protocol) in infants with retinopathy of prematurity in both treatment groups (aflibercept vs. laser photocoagulation).

Supplementary eFig. 6. Mean change from baseline in systolic and diastolic blood pressure at week 4 after baseline treatment according to baseline bodyweight in both treatment groups (aflibercept vs. laser photocoagulation).

Supplementary eFig. 7. Change from baseline to week 2 in (**a**) systolic blood pressure and (**b**) diastolic blood pressure versus concentrations of free aflibercept in plasma.

Supplementary eFig. 8. Change from baseline to week 4 in (**a**) systolic blood pressure and (**b**) diastolic blood pressure versus concentrations of adjusted bound aflibercept in plasma.

Supplementary eTable 2. Summary of aflibercept concentrations and blood pressure in infants with TESAEs occurring within 30 days of the first aflibercept injection.

Supplementary eTable 3. Summary of aflibercept concentrations and blood pressure in infants who died.

Supplementary eTable 1. Baseline demographics and characteristics of infants randomized to receive intravitreal aflibercept 0.4 mg in FIREFLEYE

	**Intravitreal aflibercept (N = 75)**
Gestational age at birth, weeks<br>Mean (SD)	26.4 (2.1)
Gestational age categories, n (%)	
<24 weeks	4 (5.3)
≥24 to <27 weeks	45 (60.0)
≥27 weeks	26 (34.7)
Chronological age at baseline, weeks<br>Mean (SD)	10.4 (2.8)
Birthweight, g<br>Median (IQR)	820.0 (640.0–1060.0)
Baseline weight, g<br>Median (IQR)	1862.0 (1580.0–2505.0)
Baseline weight range, n (%)	
800–<1000 g[a]	3 (4.0)
1000–<1500 g	11 (14.7)
1500–<2000 g	30 (40.0)
2000–<2500 g	12 (16.0)
≥2500 g	19 (25.3)
Sex, n (%)	
Female	34 (45.3)
Male	41 (54.7)
Race, n (%)[b]	
African American / Black	2 (2.7)
American Indian or Alaska Native	0
Asian	17 (22.7)
Multiple[b]	1 (1.3)
White	55 (73.3)
Country of enrollment, n (%)	
Japan	10 (13.3)
Not Japan	65 (86.7)
ROP classification[c], n (%)	
Zone I	15 (20.0)
Stage 1+	1 (1.3)
Stage 2+	2 (2.7)
Stage 3	3 (4.0)
Stage 3+	9 (12.0)
Zone II	46 (61.3)
Stage 2	0
Stage 2+	7 (9.3)
Stage 3+	39 (52.0)
AP-ROP	14 (18.7)
Zone I	12 (16.0)
Zone II	2 (2.7)
Bilateral ROP requiring treatment, n (%)	71 (94.7)

Abbreviations: AP-ROP, aggressive posterior retinopathy of prematurity; IQR, interquartile range; ROP, retinopathy of prematurity.

Data are mean (SD) unless stated otherwise.

[a]Minimum body weight at baseline (time of treatment) was 800 g; [b]Information on race was collected as part of standard demographic parameters, and classified by the investigator in fixed categories, considering information from medical records and parents; 'Multiple' describes an infant of African American/Black and White origin [c]Classification as determined at baseline by the investigator; for each infant, the ROP classification is given for the worse eye meeting inclusion criterion. Where AP-ROP or Zone were the same, the eye with the higher stage was included. The most posterior region, Zone I, is a circle with radius twice the estimated distance from the optic disc center to the foveal center. Zone II is a ring-shaped region extending nasally from the outer limit of Zone I to the nasal ora serrata and with a similar distance temporally, superiorly, and inferiorly. ROP in Zone I is more likely to progress and become severe than ROP in Zone II. Staging was defined by the appearance of a structure at the vascular–avascular juncture: demarcation line (stage 1), ridge (stage 2), and extraretinal neovascular proliferation or flat neovascularization (stage 3). If more than one ROP stage was present, the eye is classified by the most severe stage. Plus disease was defined by the appearance of dilation and tortuosity of retinal vessels. AP-ROP was used to describe a severe, rapidly progressive form of ROP located in posterior Zones I or II.

Supplementary eFig. 1. Arithmetic mean ± SD body weight through week 24 in infants with retinopathy of prematurity in both treatment groups (aflibercept vs. laser photocoagulation).

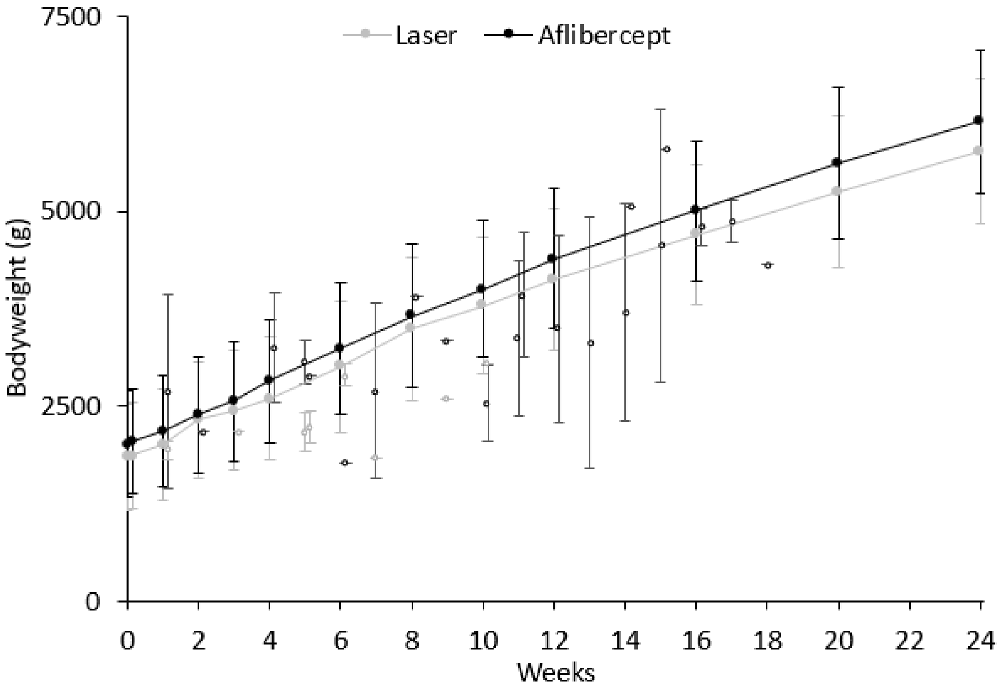

Mean ± SD weight (g)	BL	D1	W2	W4	W8	W12	W24
**Aflibercept**							
N	73	75	74	71	69	70	68
Values at visit	2022 ± 682	2053 ± 673	2389 ± 740	2819 ± 783	3661 ± 915	4393 ± 899	6148 ± 926
N		73	72	69	67	68	66
Change from BL	–	27 ± 82	364 ± 165	814 ± 277	1637 ± 462	2369 ± 545	4117 ± 755
**Laser**							
N	38	36	38	35	31	34	36
Values at visit	1851 ± 546	1868 ± 551	2322 ± 657	2599 ± 738	3492 ± 766	4127 ± 859	5765 ± 1039
N		36	38	35	31	34	36
Change from BL	–	35 ± 62	381 ± 242	781 ± 335	1586 ± 435	2278 ± 597	3941 ± 911

BL baseline, *D* day, *N* number of observations, *W* week.

The line connects data points for scheduled visits at baseline, day 1, week 1, week 2, week 3, week 4, week 6, week 8, week 10, week 12, week 16, week 20, and week 24. Data are shown for all infants, including those whose bodyweight was taken outside scheduled study visits. Week 1, day 1: *n* = 2 (aflibercept) and *n* = 2 (laser); week 2, day 1: *n* = 1 (aflibercept); week 3, day 1: *n* = 1 (laser); week 4, day 1: n = 2 (aflibercept); week 5, n = 4 (aflibercept) and n = 2 (laser); week 5, day 1: *n* = 1 (aflibercept) and *n* = 2 (laser); week 6, day 1: *n* = 1 (aflibercept) and *n* = 2 (laser); week 7: *n* = 2 (aflibercept) and *n* = 1 (laser); week 8, day 1, *n* = 1 (aflibercept); week 9: *n* = 1 (aflibercept) and *n* = 1 (laser); week 10, day 1: *n* = 3 (aflibercept) and *n* = 1 (laser); week 11: *n* = 5 (aflibercept); week 11, day 1: *n* = 3 (aflibercept); week 12, day 1: *n* = 3 (aflibercept); week 13: *n* = 2 (aflibercept); week 14: *n* = 3 (aflibercept); week 14, day 1: *n* = 1 (aflibercept); week 15: *n* = 2 (aflibercept); week 15, day 1: *n* = 1 (aflibercept); week 16, day 1: *n* = 4 (aflibercept); week 17: *n* = 4 (aflibercept); week 18: *n* = 1 (aflibercept).

Supplementary eFig. 2. Arithmetic mean ± SD concentrations of free and adjusted bound aflibercept (ng/ml) in plasma from unilaterally or bilaterally treated infants.

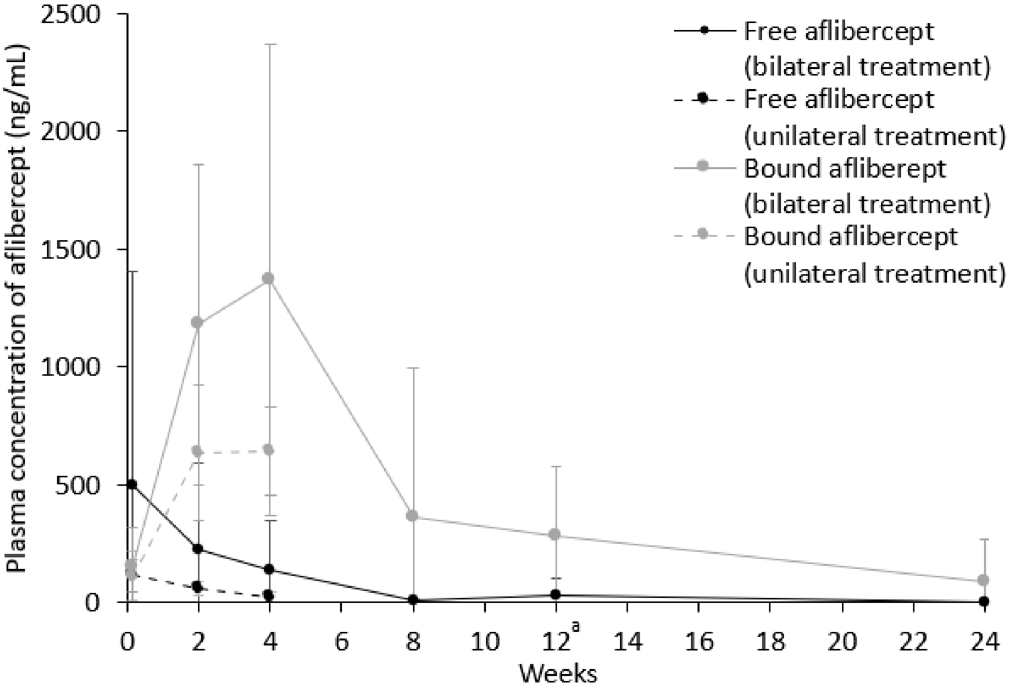

	W0/D1	W2	W4	W8	W12	W24
**Free aflibercept**						
**Unilateral treatment**						
n ≥ LLOQ / N	3/4	3/3	2/3	–	–	–
Mean ± SD (ng/mL)	113 ± 102	60.5 ± 27.9	22.3 ± 20.0	–	–	–
**Bilateral treatment**						
n ≥ LLOQ / N	63/71	57/63	55/65	1/3	1/7	0/14
Mean ± SD (ng/mL)	501 ± 905	227 ± 366	138 ± 208	5.4 ± 9.3	27.7 ± 73.3[a]	BLQ
**Adjusted bound aflibercept**						
**Unilateral treatment**						
n ≥ LLOQ / N	4/4	3/3	3/3	–	–	–
Mean ± SD (ng/mL)	112 ± 67.8	635 ± 287	643 ± 185	–	–	–
**Bilateral treatment**						
n ≥ LLOQ / N	62/71	57/62	58/64	1/3	5/7	9/14
Mean ± SD (ng/mL)	151 ± 170	1179 ± 681	1368 ± 1001	363 ± 629	281 ± 297	89.1 ± 180

D day, *LLOQ* lower limit of quantitation, *n/N* number of observations, *VEGF* vascular endothelial growth factor, *W* week.
[a]A detectable concentration of free aflibercept was measured at week 12 (194 ng/mL) in 1 infant who received aflibercept retreatment in both eyes at week 11.
Values below the LLOQ were substituted by 0 for the calculation of statistics. LLOQ was 15.6 ng/mL for free aflibercept and 31.3 ng/mL for bound aflibercept. The concentration of the bound aflibercept complex was adjusted by multiplying by 0.717 to account for the VEGF present in the bound complex (adjusted bound aflibercept).

Supplementary eFig. 3. Arithmetic mean ± SD plasma concentrations of (**a**) free and (**b**) adjusted bound aflibercept (ng/ml) in plasma, by body weight at baseline treatment

(**a**)

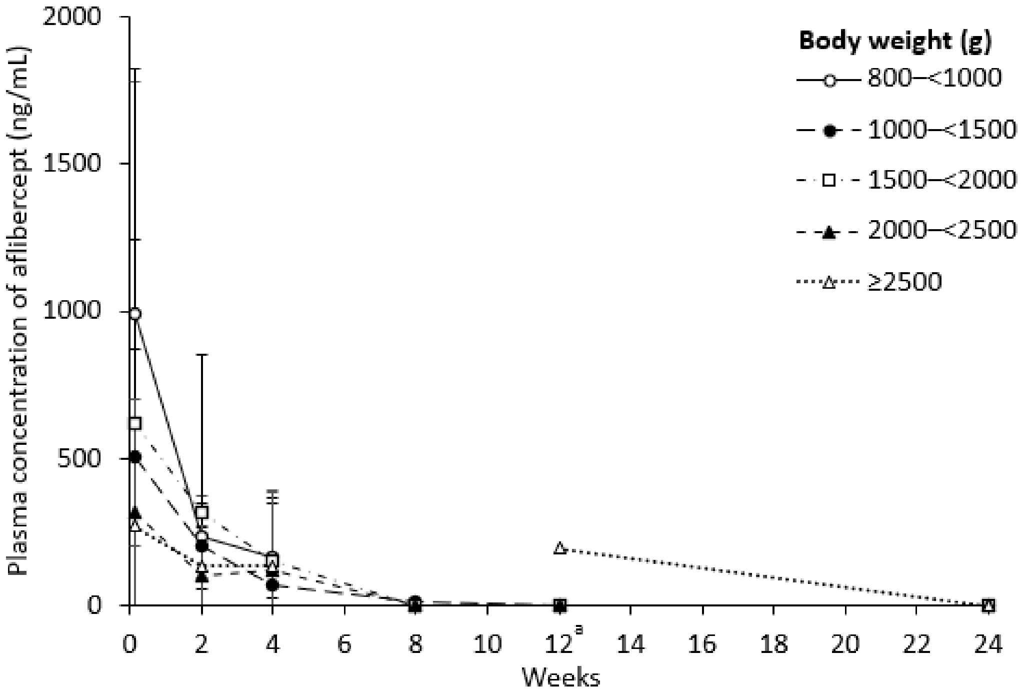

Bodyweight at treatment (g)	W0/D1	W2	W4	W8	W12	W24
**Free aflibercept**						
**800–<1000**						
n ≥ LLOQ / N	3/3	3/3	2/3			
Mean ± SD (ng/mL)	989 ± 788	233 ± 140	168 ± 227	–	–	–
**1000–<1500**						
n ≥ LLOQ / N	11/11	11/11	10/11	1/1	0/1	0/1
Mean ± SD (ng/mL)	504 ± 739	202 ± 144	68.6 ± 43.8	16.1	0	0
**1500–<2000**						
n ≥ LLOQ / N	27/30	24/26	24/29	0/1	0/3	0/7
Mean ± SD (ng/mL)	620 ± 1203	319 ± 534	155 ± 215	0	0	0
**2000–<2500**						
n ≥ LLOQ / N	9/12	7/9	7/9	0/1	0/2	0/2
Mean ± SD (ng/mL)	319 ± 552	103 ± 162	124 ± 227	0	0	0
**≥2500**						
n ≥ LLOQ / N	16/19	15/17	11/16		1/1	0/4
Mean ± SD (ng/mL)	270 ± 434	136 ± 134	136 ± 248	–	194[a]	0

D day, *LLOQ* lower limit of quantitation, *n/N* number of observations, *W* week.

[a]A detectable concentration of free aflibercept was measured at week 12 (194 ng/mL) in 1 infant who received aflibercept retreatment in both eyes at week 11.

Values below the LLOQ were substituted by 0 for the calculation of statistics. LLOQ was 15.6 ng/mL for free aflibercept.

(**b**)

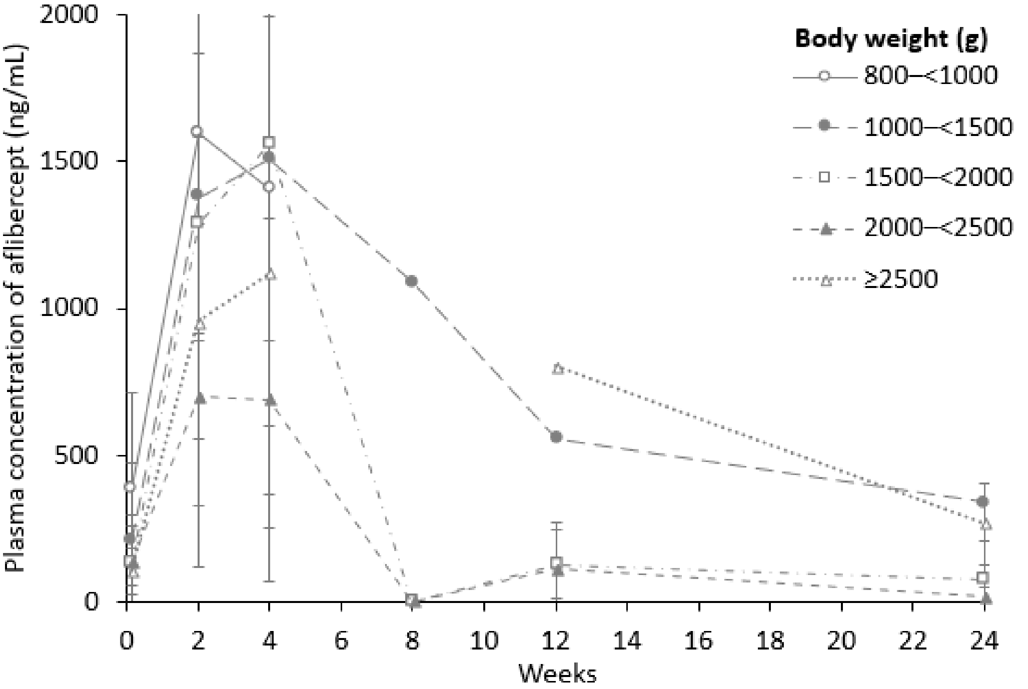

Bodyweight at treatment (g)	W0/D1	W2	W4	W8	W12	W24
**Adjusted bound aflibercept**						
**800–<1000**						
n ≥ LLOQ / N	3/3	3/3	3/3			
Mean ± SD (ng/mL)	388 ± 328	1597 ± 682	1405 ± 803	–	–	–
**1000–<1500**						
n ≥ LLOQ / N	11/11	11/11	10/11	1/1	1/1	1/1
Mean ± SD (ng/mL)	210 ± 264	1378 ± 488	1509 ± 617	1090	558	341
**1500–<2000**						
n ≥ LLOQ / N	27/30	24/26	26/29	0/1	2/3	3/7
Mean ± SD (ng/mL)	133 ± 129	1287 ± 731	1559 ± 1189	0	128 ± 117	54 ± 97
**2000–<2500**						
n ≥ LLOQ / N	10/12	7/9	7/8	0/1	1/2	1/2
Mean ± SD (ng/mL)	139 ± 160	704 ± 582	689 ± 619	0	112 ± 158	22 ± 32
**≥2500**						
n ≥ LLOQ / N	15/19	15/16	14/16		1/1	4/4
Mean ± SD (ng/mL)	106 ± 79.4	955 ± 627	1123 ± 872	–	803	269 ± 139

D day, *LLOQ* lower limit of quantitation, *n/N* number of observations, *VEGF* vascular endothelial growth factor, *W* week. Values below the LLOQ were substituted by 0 for the calculation of statistics. LLOQ was 31.3 ng/mL for bound aflibercept. The concentration of the bound aflibercept complex was adjusted by multiplying by 0.717 to account for the VEGF present in the bound complex (adjusted bound aflibercept).

Supplementary eFig. 4. Arithmetic mean ± SD plasma concentrations of (**a**) free and (**b**) adjusted bound aflibercept (ng/ml) in plasma, by gestational age at baseline treatment

(**a**)

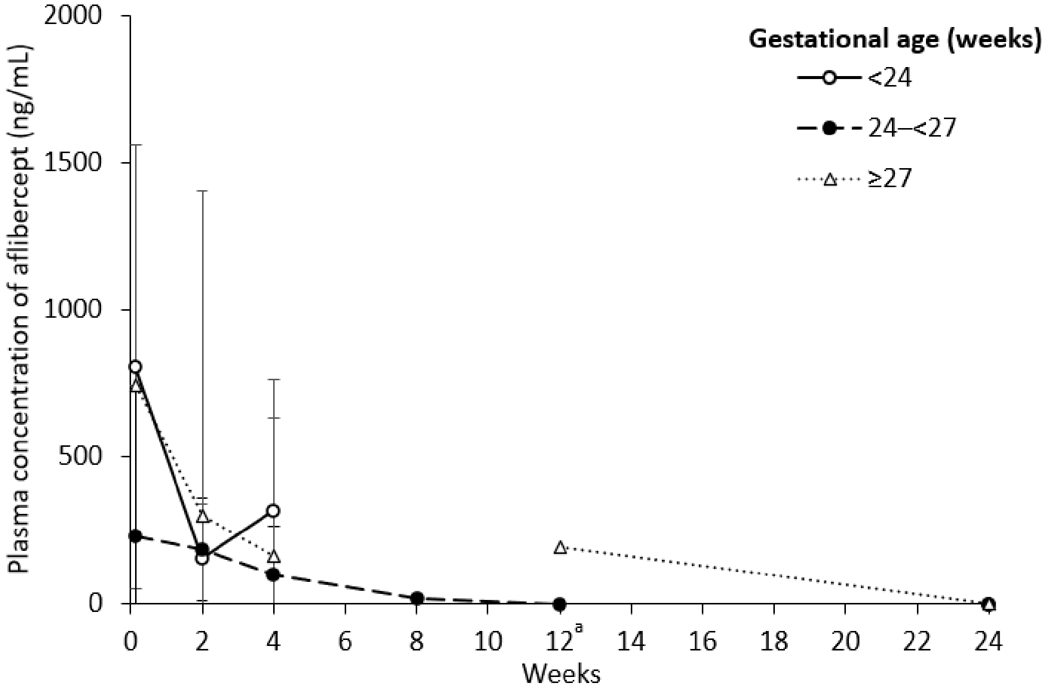

Gestational age at birth (weeks)	W0/D1	W2	W4	W8	W12	W24
**Free aflibercept**						
**<24**						
n ≥ LLOQ / N	4/4	3/3	3/4			0/1
Mean ± SD (ng/mL)	804 ± 753	156 ± 184	316 ± 316	–	–	0
**24–<27**						
n ≥ LLOQ / N	36/43	37/43	32/41	1/3	0/4	0/7
Mean ± SD (ng/mL)	229 ± 504	186 ± 173	99 ± 164	16.1	0	0
**≥27**						
n ≥ LLOQ / N	24/26	20/20	19/23		1/3	0/6
Mean ± SD (ng/mL)	742 ± 1106	299 ± 601	163 ± 238	–	194[a]	0

D day, *LLOQ* lower limit of quantitation, *n/N* number of observations, *W* week.
[a]A detectable concentration of free aflibercept was measured at week 12 (194 ng/mL) in 1 infant who received aflibercept retreatment in both eyes at week 11.
Values below the LLOQ were substituted by 0 for the calculation of statistics. LLOQ was 15.6 ng/mL for free aflibercept.

(**b**)

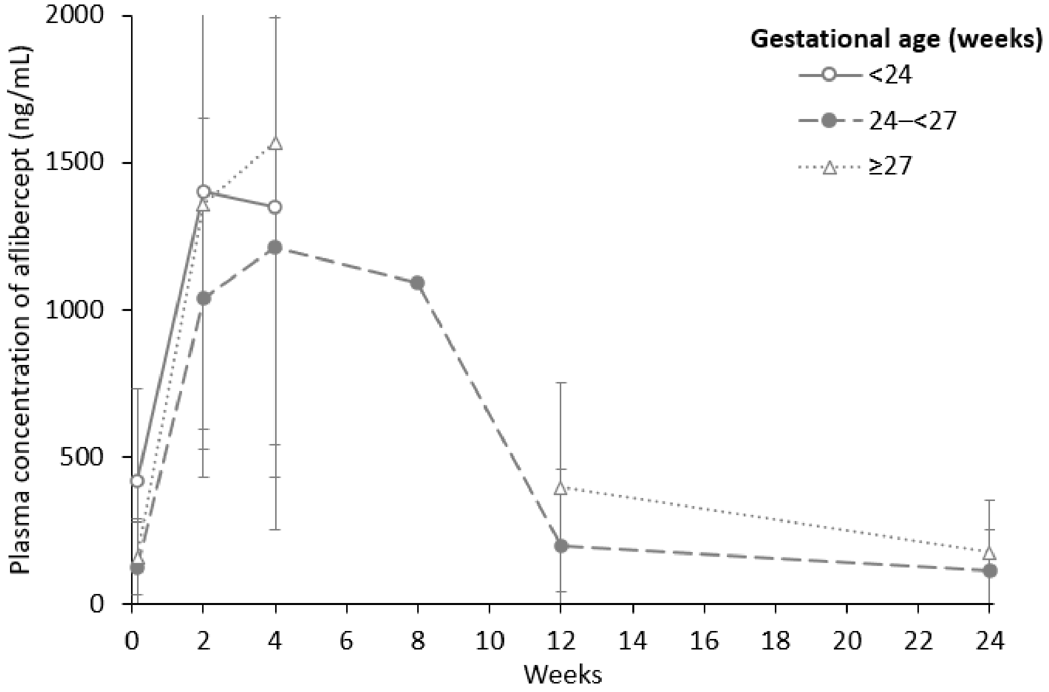

Gestational age at birth (weeks)	**W0/D1**	**W2**	**W4**	**W8**	**W12**	**W24**
**Adjusted bound aflibercept**						
**<24**						
n ≥ LLOQ / N	4/4	3/3	4/4			0/1
Mean ± SD (ng/mL)	419 ± 310	1402 ± 877	1347 ± 808	–	–	0
**24–<27**						
n ≥ LLOQ / N	37/43	37/42	36/41	1/3	2/4	4/7
Mean ± SD (ng/mL)	123 ± 153	1040 ± 609	1210 ± 782	1090	195 ± 264	113 ± 141
**≥27**						
n ≥ LLOQ / N	24/26	20/20	21/22		3/3	5/6
Mean ± SD (ng/mL)	159 ± 129	1357 ± 763	1568 ± 1317	–	396 ± 355	175 ± 174

D day, *LLOQ* lower limit of quantitation, *n/N* number of observations, *VEGF* vascular endothelial growth factor, *W* week.Values below the LLOQ were substituted by 0 for the calculation of statistics. LLOQ was 31.3 ng/mL for bound aflibercept. The concentration of the bound aflibercept complex was adjusted by multiplying by 0.717 to account for the VEGF present in the bound complex (adjusted bound aflibercept).

Supplementary eFig. 5. Arithmetic mean ± SD systolic and diastolic blood pressure through week 24 (including visits outside those scheduled in the protocol) in infants with retinopathy of prematurity in both treatment groups (aflibercept vs. laser photocoagulation).

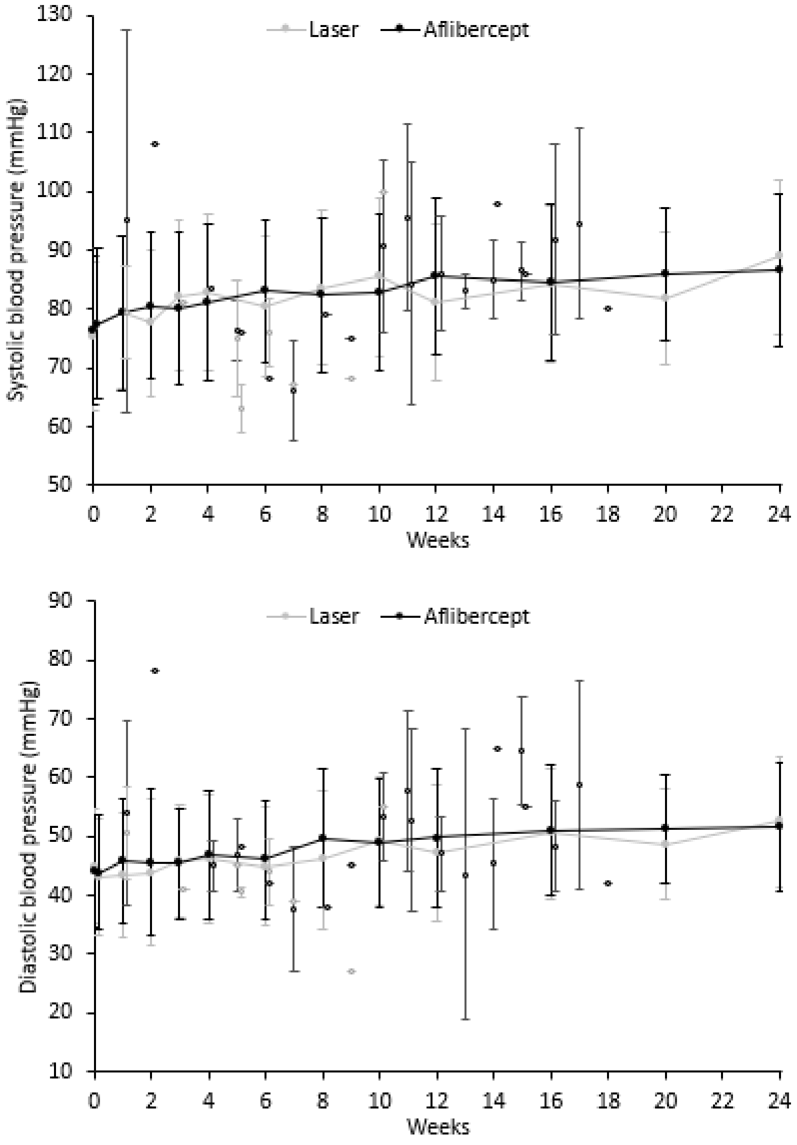

BL baseline, *D* day, *N* number of observations, *W* week.

The line connects data points for scheduled visits at baseline, day 1, week 1, week 2, week 3, week 4, week 6, week 8, week 10, week 12, week 16, week 20, and week 24. Data are shown for all infants including those whose blood pressure was taken outside scheduled study visits. Week 1, day 1: *n* = 2 (aflibercept) and *n* = 2 (laser); week 2, day 1: *n* = 1 (aflibercept); week 3, day 1: *n* = 1 (laser); week 4, day 1: *n* = 2 (aflibercept); week 5, *n* = 4 (aflibercept) and *n* = 2 (laser); week 5, day 1: *n* = 1 (aflibercept) and *n* = 2 (laser); week 6, day 1: *n* = 1 (aflibercept) and *n* = 2 (laser); week 7: *n* = 2 (aflibercept) and *n* = 1 (laser); week 8, day 1, *n* = 1 (aflibercept); week 9: *n* = 1 (aflibercept) and *n* = 1 (laser); week 10, day 1: *n* = 3 (aflibercept) and *n* = 1 (laser); week 11: *n* = 5 (aflibercept); week 11, day 1: *n* = 3 (aflibercept); week 12, day 1: *n* = 3 (aflibercept); week 13: *n* = 2 (aflibercept); week 14: *n* = 3 (aflibercept); week 14, day 1: *n* = 1 (aflibercept); week 15: *n* = 2 (aflibercept); week 15, day 1: *n* = 1 (aflibercept); week 16, day 1: *n* = 4 (aflibercept); week 17: *n* = 4 (aflibercept); week 18: *n* = 1 (aflibercept).

Supplementary eFig. 6. Mean change from baseline in systolic and diastolic blood pressure at week 4 after baseline treatment according to baseline bodyweight in both treatment groups (aflibercept vs. laser photocoagulation).

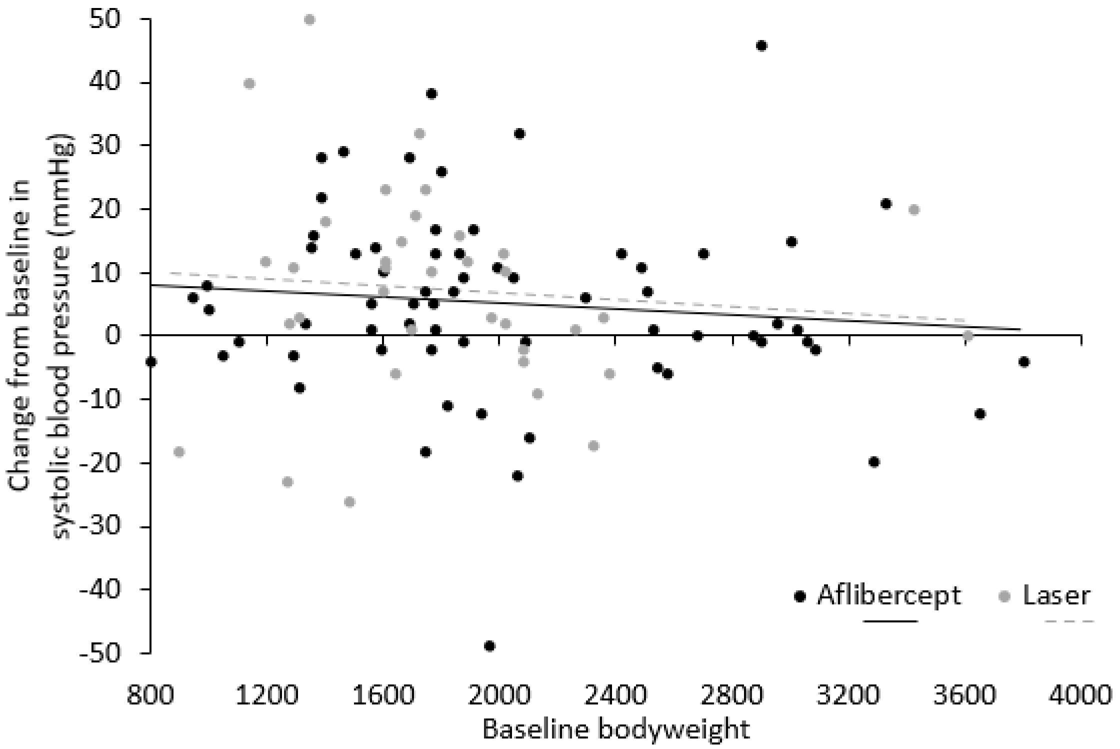

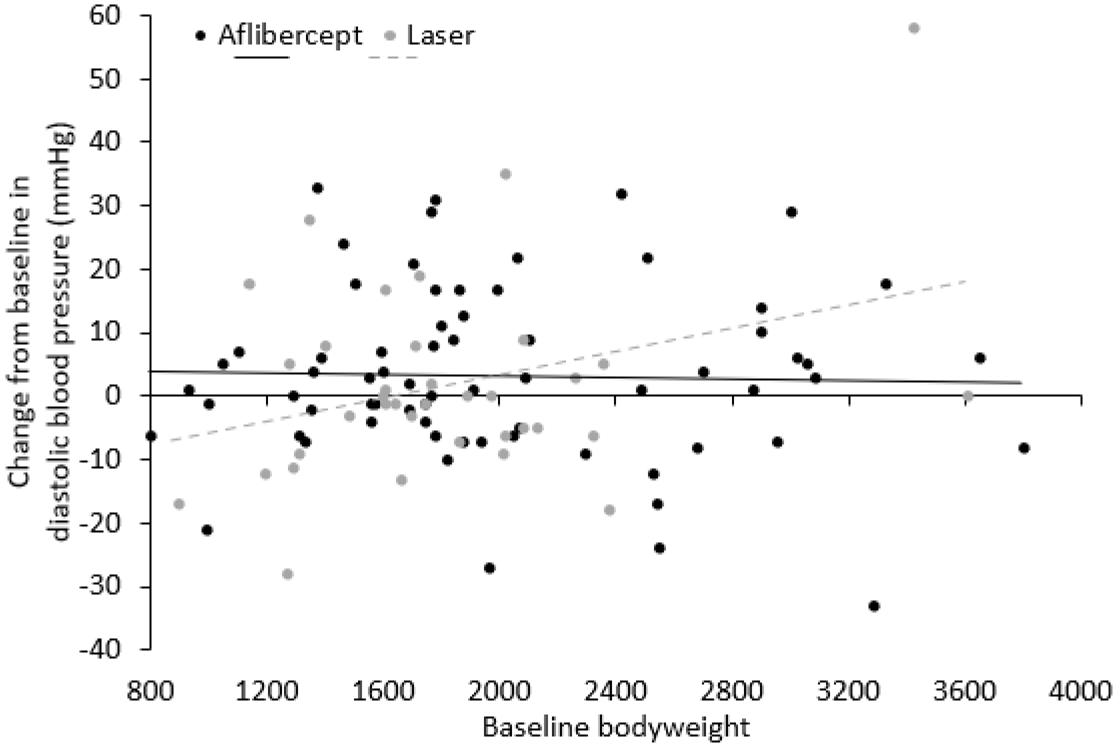

Individual observations in the aflibercept and laser groups are shown by black and gray circles, respectively. The dotted line depicts the regression line for laser; the solid line, the regression line for aflibercept.

Supplementary eFig. 7. Change from baseline to week 2 in (**a**) systolic blood pressure and (**b**) diastolic blood pressure versus concentrations of free aflibercept in plasma.

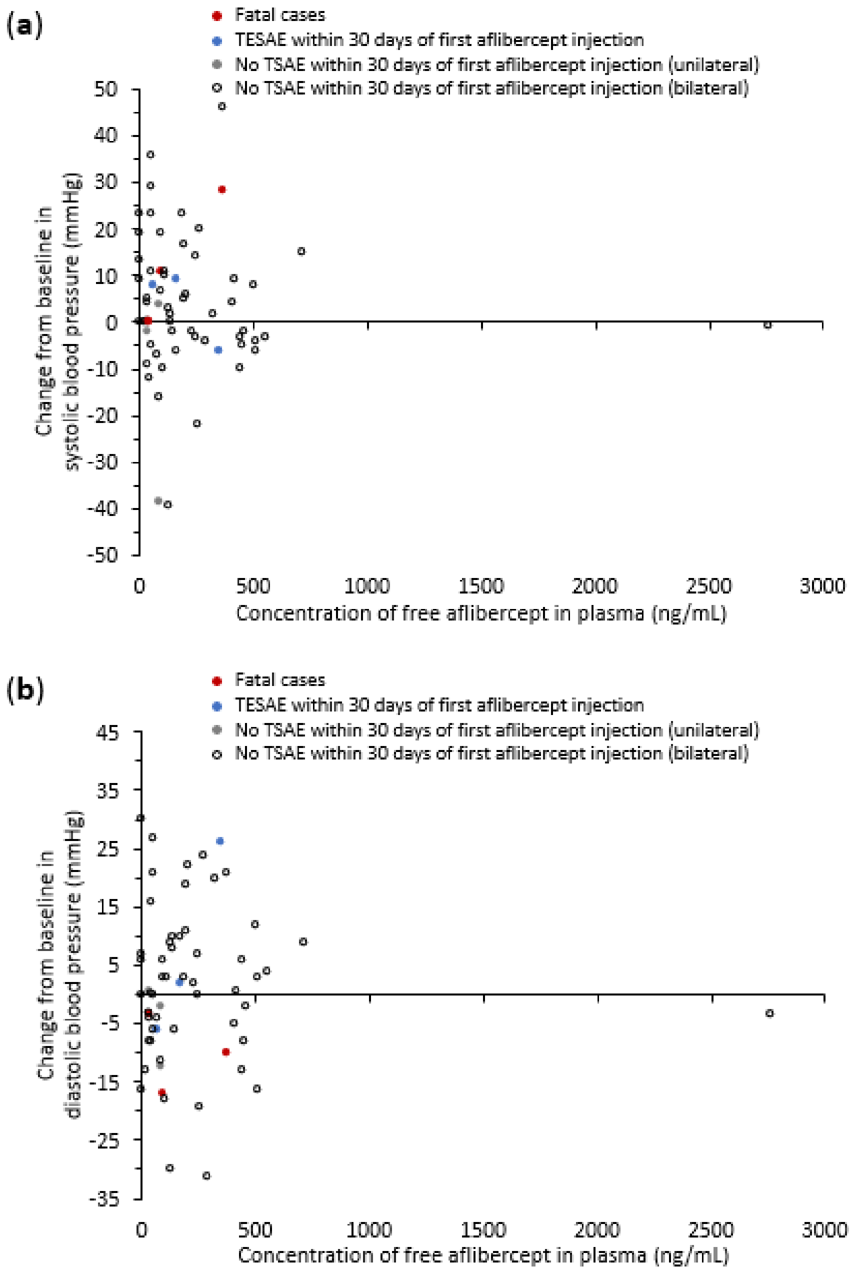

LLOQ lower limit of quantitation, *TESAE* treatment-emergent serious adverse event, *VEGF* vascular endothelial growth factor. Infants with TESAEs during the first 30 days following the start of treatment who had missing values for either aflibercept plasma concentrations and/or blood pressure values at single time points are not included in the figure. Values below the LLOQ were substituted by 0. LLOQ was 15.6 ng/mL for free aflibercept.

Supplementary eFig. 8. Change from baseline to week 4 in (**a**) systolic blood pressure and (**b**) diastolic blood pressure versus concentrations of adjusted bound aflibercept in plasma

(a)

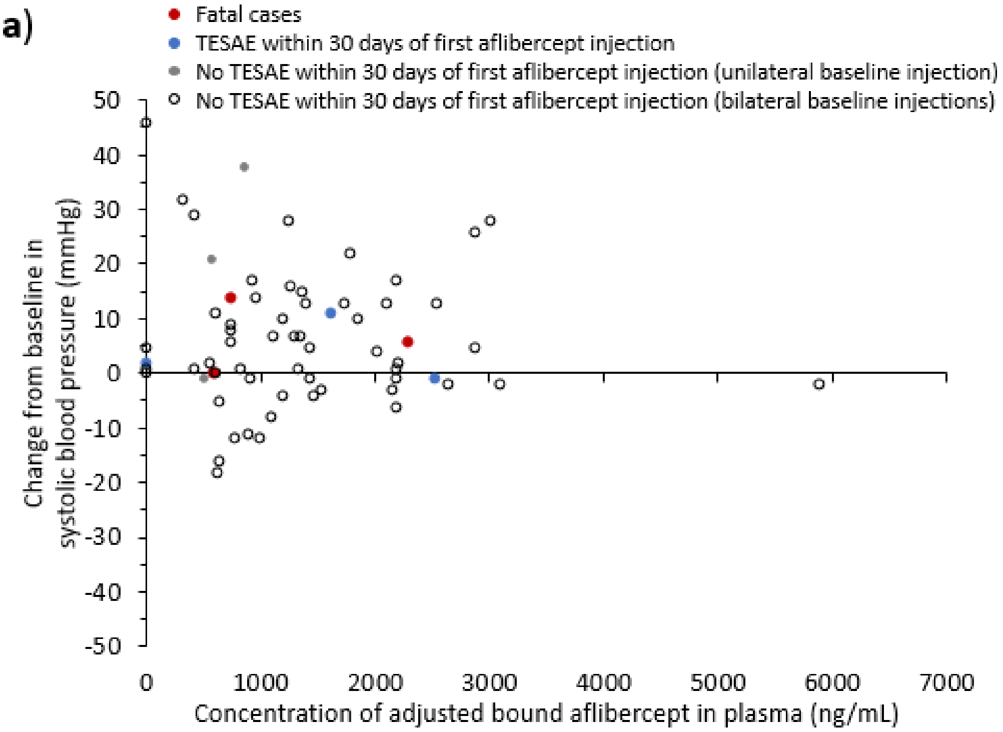

(b)

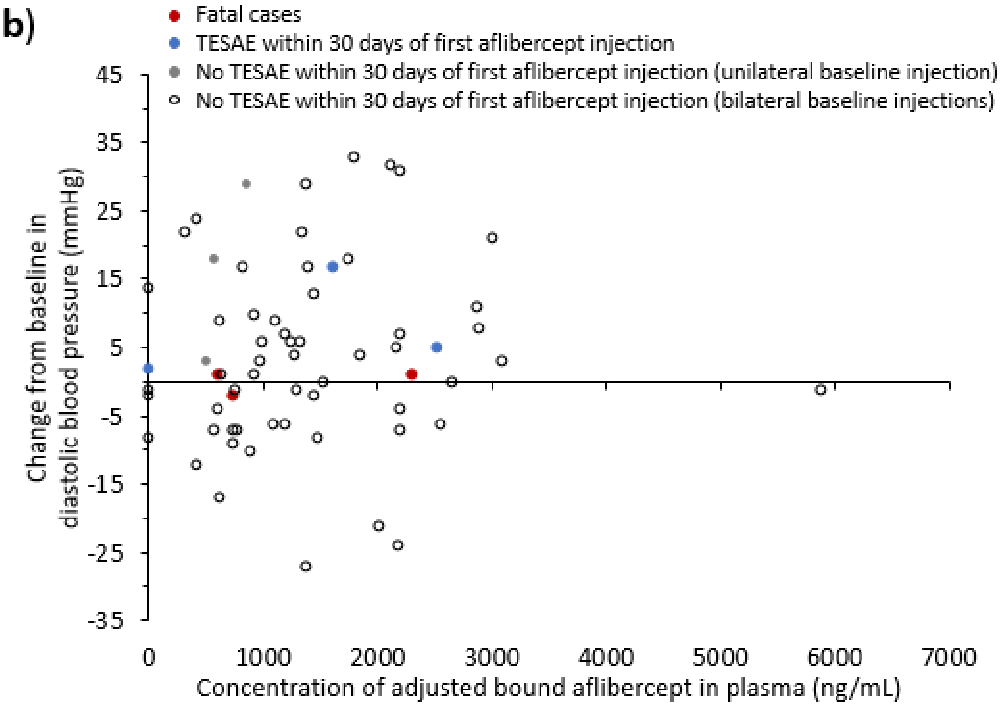

LLOQ lower limit of quantitation, *TESAE* treatment-emergent serious adverse event, *VEGF* vascular endothelial growth factor.Infants with TESAEs during the first 30 days following the start of treatment who had missing values for either aflibercept plasma concentrations and/or blood pressure values at single time points are not included in the figure. Values below the LLOQ were substituted by 0. LLOQ was 31.3 ng/mL for bound aflibercept. The concentration of the bound aflibercept complex was adjusted by multiplying by 0.717 to account for the VEGF present in the bound complex (adjusted bound aflibercept).

Supplementary eTable 2. Summary of aflibercept concentrations and blood pressure in infants with TESAEs occurring within 30 days of the first aflibercept injection.

	**TESAE details**			**Concentrations of aflibercept in plasma**			**Vital signs on pharmacokinetic sampling days**		
**TESAE (preferred term)**	**TESAE onset / stop (study day)**	**TESAE severity**	**Outcome**	**Day**	**Free aflibercept (ng/mL)**	**Adjusted bound aflibercept (ng/mL)**	**SBP (mm Hg)**	**DBP (mm Hg)**	**Weight (kg)**
Pneumonia aspiration	28 / 33	Moderate	Recovered/ resolved	2<br>15<br>36	2380<br>160<br>105	377<br>1649<br>1606	75<br>79<br>81	38<br>38<br>53	1.940<br>2.250<br>3.010
Retinal detachment	16 / –	Severe	Not recovered/ not resolved	2<br>30	<15.6<br><15.6	<22.4<br><22.4	68<br>74	35<br>42	1.690<br>2.320
COVID-19 and pneumonia	23 / 48	Mild	Recovered/ resolved	2<br>13<br>55	34<br>351<br>329	42.2<br>997<br>1090	114<br>115<br>N/A	56<br>94<br>N/A	3.431<br>3.628<br>N/A
(Worsening of) retinopathy of prematurity	14 / –	Moderate	Recovering/ resolving	4<br>14	115<br>64.5	152<br>620	N/A<br>75	N/A<br>44	N/A<br>2.175
Intraocular pressure increased, corneal edema and overdose[a]	1 / 2	Moderate	Recovered/ resolved	2<br>14<br>33<br>165	65.5<br>26.2<br>38.4<br><15.6	149<br>2129<br>2517<br>288	75<br>74<br>74<br>77	44<br>45<br>49<br>49	3.050<br>3.600<br>4.560<br>8.100
Bronchiolitis	5 / 30	Moderate	Recovered/ resolved	2	1140	176	87	48	2.310
**Concentrations of aflibercept in plasma in patients without TESAE for comparison**				**1<br>14<br>28<br>56<br>84<br>168**	**<15.6 to 4570<br><15.6 to 2750<br><15.6 to 923<br><15.6 to 16.1<br><15.6 to 194[b]<br><15.6**	**<22.4 to 968<br><22.4 to 2646<br><22.4 to 5887<br><22.4 to 1090<br><22.4 to 803<br><22.4 to 457**			

COVID-19 coronavirus disease 2019, *DBP* diastolic blood pressure, *LLOQ*, lower limit of quantitation, *N/A* not available, *SBP* systolic blood pressure, *TESAE* treatment-emergent serious adverse event, *VEGF* vascular endothelial growth factor.

[a]Drug overdose in 1 eye of a bilaterally injected patient with transient increase in intraocular pressure and corneal edema.

[b]A detectable concentration of free aflibercept was measured at week 12 (194 ng/mL) in an infant who received aflibercept re-treatment in both eyes at week 11.

LLOQ was 15.6 ng/mL for free aflibercept and 31.3 ng/mL for bound aflibercept. The concentration of the bound aflibercept complex was adjusted by multiplying by 0.717 to account for the VEGF present in the bound complex (adjusted bound aflibercept).

Supplementary eTable 3. Summary of aflibercept concentrations and blood pressure in infants who died.

TESAE details			Aflibercept plasma concentrations			Vital signs on pharmacokinetic sampling days		
**TESAE (preferred term)**	**Interval between baseline treatment and death (days)**	**Interval between last treatment and death (days)**	**Day**	**Free aflibercept (ng/mL)**	**Adjusted bound aflibercept (ng/mL)**	**SBP (mm Hg)**	**DBP (mm Hg)**	**Weight (kg)**
Bronchopulmonary dysplasia, pneumothorax	144	59	3<br>17<br>29	1690<br>365<br>426	746<br>2380<br>2294	80<br>90<br>68	41<br>33<br>44	0.936<br>1.109<br>1.386
Bronchiolitis	57	56	2<br>14<br>29	515<br>85.5<br>26	214<br>796<br>731	79<br>96<br>99	43<br>40<br>55	1.400<br>1.750<br>2.240
Bronchopulmonary dysplasia	61	28	4<br>18<br>29	112<br>37.1<br>444	225<br>516<br>591	85<br>84<br>84	48<br>45<br>49	2.851<br>3.234<br>3.480

DBP diastolic blood pressure, *LLOQ*, lower limit of quantitation, *SBP* systolic blood pressure, *TESAE* treatment-emergent serious adverse event, *VEGF* vascular endothelial growth factor.

LLOQ was 15.6 ng/mL for free aflibercept and 31.3 ng/mL for bound aflibercept. The concentration of the bound aflibercept complex was adjusted by multiplying by 0.717 to account for the VEGF present in the bound complex (adjusted bound aflibercept).
Deaths were assessed by the investigator as causally unrelated to study drug.
